# A Review of Emotion Recognition Methods Based on Data Acquired via Smartphone Sensors

**DOI:** 10.3390/s20216367

**Published:** 2020-11-08

**Authors:** Agata Kołakowska, Wioleta Szwoch, Mariusz Szwoch

**Affiliations:** Faculty of Electronics, Telecommunications and Informatics, Gdańsk University of Technology, 80-233 Gdansk, Poland; wszwoch@eti.pg.edu.pl (W.S.); szwoch@eti.pg.edu.pl (M.S.)

**Keywords:** affective computing, emotion recognition, smartphones, sensory data, sensors, human–computer interaction

## Abstract

In recent years, emotion recognition algorithms have achieved high efficiency, allowing the development of various affective and affect-aware applications. This advancement has taken place mainly in the environment of personal computers offering the appropriate hardware and sufficient power to process complex data from video, audio, and other channels. However, the increase in computing and communication capabilities of smartphones, the variety of their built-in sensors, as well as the availability of cloud computing services have made them an environment in which the task of recognising emotions can be performed at least as effectively. This is possible and particularly important due to the fact that smartphones and other mobile devices have become the main computer devices used by most people. This article provides a systematic overview of publications from the last 10 years related to emotion recognition methods using smartphone sensors. The characteristics of the most important sensors in this respect are presented, and the methods applied to extract informative features on the basis of data read from these input channels. Then, various machine learning approaches implemented to recognise emotional states are described.

## 1. Introduction

Nowadays, smartphones and other mobile devices accompany us in our daily life, and a world without them seems unrealistic and unlikely. In the beginning, mobile phones offered mobility and voice calls, but they are no longer just tools used to talk, as they have transformed into much more powerful devices [1]. In many cases, they replace computers due to their small size, mobility, and because they are equipped with many possibilities. One can use them to search for information on the web, check e-mails, play games, download multimedia files, read e-books, take good quality photos and video recordings, listen to music, navigate, and much more. A high-resolution colour display, wireless network support, music players, large memory, etc. have become standard nowadays. Many functions of modern smartphones are made possible by using one or more built-in sensors. Most devices include a built-in camera, GPS, accelerometer, gyroscope, magnetometer, and others. These sensors provide important information to many advanced applications in different areas such as entertainment, education, virtual (VR), augmented (AR) and mixed reality (MR), health monitoring, etc. One of the possible applications that is attracting significant attention nowadays is emotion recognition. Affective mobile applications fall into the category of mobile sensing, either personal when it focuses on monitoring personal information or social when the information is shared within a group [2].

The aim of this paper is to provide a comprehensive and systematic review of emotion recognition methods and approaches based on the different sensory data available on modern smartphones. Several review studies on mobile affect recognition have already been presented. Some of them take into account selected emotional states, e.g., happiness [3], while some focus on selected input modalities, e.g., in [4], where the authors present a general point of view but also focus on the difficulties faced while sensing affect from facial expression and voice. An extensive and valuable review has been presented in [5], where the full spectrum of research studies published between 2010 and 2017 has been presented.

**Figure 1:**
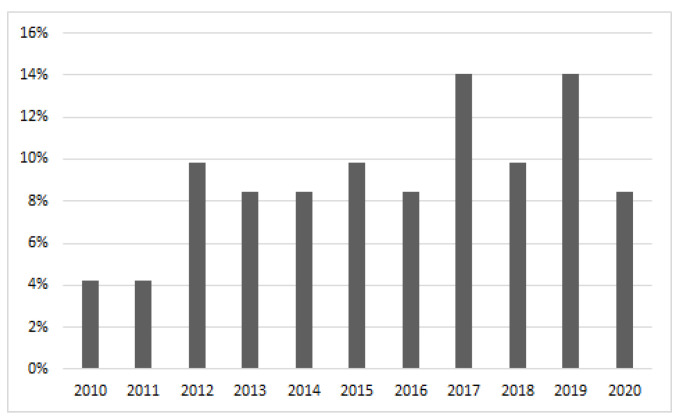
Distribution of reviewed papers across the selected time span (for 2020, only the first half of the year was taken into account).

This review is based on papers published since 2010. The aim of the research was to identify papers focusing on the recognition of emotions or mood on the basis of data recorded via smartphone sensors. We intended to put emphasis on representation of data from various sources, feature extraction, and selection, which require different approaches depending on the source. Therefore, a decision was made to exclude the camera and microphone from the investigation, as a thorough review of methods based on these two sensors could be a separate, extensive survey. In the end, the selected papers were supposed to take into account at least one of the following input channels: touchscreen, accelerometer, gyroscope, magnetometer, light sensor, GPS, Bluetooth. Additionally, a study could also incorporate other data taken only using a smartphone, e.g., noise level recorded via the microphone, phone usage measured by calls, messages, and applications running. On the other hand, any study requiring the use of other than phone devices, e.g., smart bands, were excluded from the survey. Moreover, numerous studies on monitoring mental disorders, which relate to mood monitoring but make a separate characteristic group, were also excluded. After the first search phase, the abstracts were looked through, which eventually led to us selecting 102 papers. Some of them were rejected on the basis of the content of the paper. Moreover, a review of the references of the selected papers led to us including a number of publications previously not found. Eventually, 75 papers were thoroughly reviewed. Figure 1 presents the distribution of papers selected for the review. The histogram, to a certain extent, shows a continuous development in methods, tools, and applications designed in the area of emotion recognition based on data from smartphone sensors.

The article is organised as follows. In Section 2, a brief introduction to the affective computing and emotion recognition domains is given. In Section 3, the most popular smartphone sensors are described. Section 4 presents the reviewed methods from the point of view of data collection and labelling, data representation, feature selection, model training, and recognition accuracy. Section 5 summarises the survey.

## 2. Emotion Recognition

Emotions accompany people throughout their lives and play an important role, influencing both their physiological and mental states. The ability to recognise the emotional state of another person is very important from the point of view of interpersonal relations and communication. In recent years, there has been increasing interest in the automatic recognition of emotions and taking into account the emotional state of a person in various applications in many fields. Solutions implementing the automated recognition of emotions are used, among others, in marketing, systems monitoring driver tiredness, e-learning, healthcare, education [6], entertainment [7,8], software engineering [9,10], etc. Many different approaches to emotion recognition have been developed, which use more and more advanced algorithms achieving increasing effectiveness. Optimisation of solutions allows for their implementation with the use of everyday equipment such as mobile devices. Currently, the commonly available devices have sufficient computing power and enable the use of various signals such as image, sound, heart rate measurement, and other sensory data.

There are many ways of expressing emotions, including visual, verbal, behavioural, physiological, and others. In general, it can be assumed that the first three types of emotional expression are subject to greater user control, and the degree and frequency of expressing them may be significantly influenced by both the personality type and cultural and social factors as well as the current environment of a given person. This is understandable because the visual and verbal channels are the main way of expressing emotions in interpersonal relationships. On the other hand, it is much more difficult for people to control their physiological reactions related to the expression of certain emotions. Unfortunately, a significant obstacle from the point of view of automatic recognition is the high variability of biosignals, not only individual, but also dependent on the current situation of a given person.

Facial expressions (FE) are one of the main information channels in interpersonal communication. Through facial expressions, we are able to express (and recognise) many different shades of emotions. Since facial expressions are the result of the work of facial muscles, it is possible to formally represent them in a form of facial action coding system (FACS) [11]. Currently, many libraries and applications offer automatic emotion recognition using static camera images or video recordings. A person also expresses emotions by using gestures and body posture. For automatic recognition of emotions, body language is much more challenging than facial expression analysis.

Numerous studies have been conducted on automatic facial expression recognition (FER) due to its practical importance in many application fields. In addition, several exhaustive surveys on FER have been published in recent years. They include both standard recognition pipelines for FER [12,13,14,15] as well as approaches based on deep learning [16,17]. These articles and surveys include both static image FER based on spatial features as well as dynamic dynamic-based methods which take into account also temporal relations between subsequent frames in the input facial expression sequence. Survey articles also contain lists and categorisation of the features used, available databases, and data sets, as well as the learning algorithms and classifiers used [16]. In recent years, there has also been a growing interest in recognising emotions based on human gestures, posture, and gait. As emotion recognition using these modalities is generally less effective than FER, many researchers are using multimodal approaches for one or more information channels and developing early and late fusion methods. A survey of publications in this domain can be found, among others in [18,19,20,21].

The verbal channel is as important an element of emotional expression as the visual channel. It plays an increasingly important role in interpersonal communication due to the ever growing share of remote communication using only the voice channel. In the absence of an interlocutor’s image, emotions can be expressed by word choice, tone, loudness and style of speech, as well as other nuances. Recognising emotions based on voice becomes especially important in the era of widespread use of personal voice assistants such as Amazon Alexa, Google Assistant, Apple Siri, and Microsoft Cortana, which are widely available in smartphones, smart watches, and speakers [22]. Numerous publications in this field are collected through extensive surveys, including [23,24,25]. Since the audio channel does not provide continuity in recognising emotions, and the effectiveness of recognition depends on many factors, including voice quality, this source of information is very often used in multimodal systems [26,27,28]. For example, an interesting *emotographic model* analyses indicators residing in standard multimodal data produced by commonly used applications and Internet of things (IoT) devices to interpret human emotional state [22].

Emotions felt by people are also reflected in their biosignals such as heart rate, skin conductance, blood volume pulse, EEG, muscle tension, respiration, and others [29]. Physiological signals are generally an objective source of information about real human emotions because they are fundamentally independent of our will and, unlike facial expressions, we cannot control them.

Many different models of emotion have been proposed, which can generally be categorised as discrete, continuous, and componential [30,31]. Discrete models, the most important of which are Ekman’s and Plutchik’s, contain a literal list of recognised emotions. Continuous models allow the presentation of emotions in one, two, or three-dimensional coordinate systems, the axes of which are most often pleasure (P) or valence (V), arousal (A), and dominance (D). Although the PAD/VAD and PA/VA models are the most popular, in many affective applications, especially in the field of education and entertainment, simplified models are used for one of the selected values, i.e., active, neutral, and passive state (A). In turn, in componential models, an example of which is the Ortony, Clore, and Collins (OCC) model, use several factors that create or influence the emotional state. The OCC model takes into account the process of generating emotions and allows you to predict what emotion will arise in response to events, actions, or objects, which is why it makes this model suitable for use in artificial agents.

Scientific research in the field of emotion recognition most often uses one of two models of emotions and their extension or simplification-Ekman’s and PAD/VAD. Research by the American psychologist Paul Ekman on human emotions and facial expressions allowed a set of six basic emotions to be identified, e.g., happiness, surprise, anger, fear, sadness, and disgust, which are intercultural and universally recognised regardless of age, gender, or external conditions [11]. Most studies most often involve an additional neutral state. In many cases, some basic emotions are overlooked or combined with others because of difficulties in recognising or distinguishing them.

**Figure 2:**
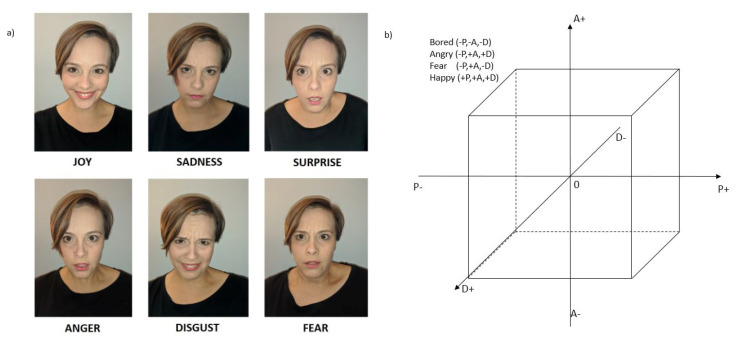
(**a**) Sample illustrations of six Ekman’s emotions (**b**) Sample representation of PAD model.

In the PAD/VAD model, emotions are placed in a three-dimensional PAD/VAD space (Figure 2). The pleasure/valence determines whether an emotion is pleasant or unpleasant to the person, that is, it distinguishes between positive and negative emotions. The arousal differentiates between active and passive emotions. The dominance represents the reaction of fight or flight, indicating the controlling and dominant nature of emotion. Zero point values of the coordinate system are assigned an emotionally neutral state. Each emotion can be represented as a linear combination of those three components, e.g., anger can be defined near (−0.51, 0.59, 0.25) point in the PAD/VAD coordination system. The main problem of continues models is that it is not natural for people to define their emotions by numbers, especially decomposed into independent factors. In addition, such a description is not accurate, and the values given are averages, so they may differ for individual people.

In recognising emotions, one has to consider the choice of emotional model. This depends mostly on the application. For example, it will be important to detect boredom and stress in e-learning applications, while joy and sadness will be more appropriate for entertainment models. It is not always necessary to recognise specific emotions. In some applications, pleasure or arousal (PA) detection is sufficient.

**Figure 3:**
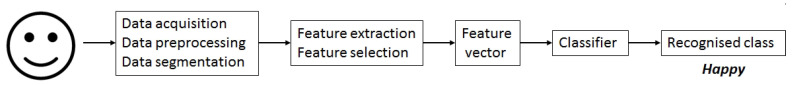
General emotion recognition process.

In general, the process of recognising emotions is similar to the standard pattern recognition schema. The methods of recognising emotions can be divided into two main categories. The first assumes the use of conventional machine learning methods, which require elements precisely designed by man (Figure 3). The second category includes deep learning, which is capable of improving their capabilities without human intervention.

In the first step, the data is acquired and preprocessed in a way that depends on the signal we use. For example, for images, it can be noise removal, sharpening, contrast improvement, brightness normalisation, etc. The segmentation process is optional; for images, it can be, for instance, background removal, face detection, size normalisation. The next step is to select a set of features. If this set is very large, the feature selection can be made to chose the most informative. Using the deep learning approach, this set is much simpler and even removed, as this group of methods can operate on raw data. The final step is classification and assigning data to one of the possible classes. To recognise emotions, classification methods commonly known from other fields are used. In the literature, you can find, for example, Support Vector Machine (SVN), kNearest Neighbor (k-NN), naïve Bayes, Discriminant Function Analysis, Fisher Linear Discriminant Analysis (LDA), Discriminant Analysis (FDA), and many others. Deep learning does not require specific features. In this case, however, we need a relatively large data set to train generalisation skills. If the available data set is too small, its size can be enlarged artificially by data augmentation methods.

## 3. Smartphone Sensors and Input Channels for Emotion Recognition

Modern smartphones, despite the obvious telecommunication functions, play the role of mobile personal computers, media, and edutainment centres and provide the user with high speed Internet access and various utility and tool applications. They are also usually equipped with several input devices and many sensors that provide different information channels about the users and their environment. Such rich, multimodal information enables smartphones to offer their users many additional and advanced capabilities including navigation, game control, virtual, augmented and mixed reality, emotion recognition, and many others. In fact, these sensors are what make mobile devices really smart and helpful.

Starting with a microphone and the camera, the number of sensors available in smartphones has steadily increased in recent years, including, for example, accelerometer, gyroscope, compass, GPS, light, temperature, proximity, pressure sensors, and others. Thanks to them, smartphones, for example, can count the user’s steps, knows its localisation and spatial orientation, automatically adjusts the screen brightness, etc. Different biosensors allow the user to secure access to the device, and some of them to scan the user’s face, record body movements, control gestures, or recognise facial expressions.

Some sensors are built into smartphones using different miniaturised technologies, such as microelectromechanical systems (MEMS). In this technology, very tiny mechanical systems are embedded into a tiny electronic chip [32]. In addition to built-in sensors, there are external sensors that can communicate with smartphones [1,33]. Sensors in the close surroundings of the phone transmit data using, for instance, a WiFi or Bluetooth connection. Examples include a temperature, humidity, and altimeter sensor. The sensors used in smartphones can also cooperate, resulting in new functionalities or improvements. For example, the camera can use the accelerometer or gyroscope to determine portrait vs. landscape mode and also to provide more advanced stabilisation [1].

Sensors can be classified according to different criteria. For example, they can be divided into *physical* which are embedded directly into smartphones (e.g., accelerometer, gyroscope) and *virtual*-*software-based* sensors deriving their data from physical sensors (e.g., gravity sensors) [1]. Another classification divides them into *motion* (e.g., accelerometer, gyroscope), *environment* (e.g., camera, depth sensors), and *position* (e.g., GPS) sensors [34].

While recognising emotions is not the main task of smartphones, their current computing power (or access to cloud computing) and numerous input devices and sensors allow them to successfully accomplish this task. The sensors and input devices that are most important for emotion recognition are presented next.

### 3.1. Camera

Cameras were introduced to mobile phones very early. Initially in the form of external extensions, they quickly became an important built-in optical sensor. Initially, their low resolution and sensitivity as well as poor optics severely limited their use to taking low-quality photos. However, the progressive miniaturisation and improvement of the quality of photosensitive matrices and lenses allowed for the development of an increasing number of applications, ranging from amateur photography, through scanning and processing two-dimensional documents (camera-based document analysis and recognition, CBDAR), and ending with advanced analysis of the three-dimensional environment, allowing for the scanning and reconstruction of 3D objects (multiview stereo, MVS), indoor and outdoor mapping and navigation systems (I/O MMS, simultaneous localisation and mapping, SLAM), motion and facial expression capture systems, and many others. In fact, it was the development of smartphone cameras that resulted in the elimination of compact cameras from the market, and for several years, a lot of amateurs (and even some professional photographers) have been willing to use smartphones to document their lives instead of SLRs or mirrorless cameras.

Modern smartphones generally include cameras on both sides. More advanced mid-to-high-end designs typically have several rear (back) cameras, such as standard, wide, and tele ones. For example, the Apple iPhone 11 Pro Max uses a triple-camera system with 12MP sensors [35]. Rear cameras are usually of much better quality (bigger and more sensitive sensors, glass instead of plastic lens, optical stabilisation systems, etc.) and can be used in the most advanced applications. Unfortunately, due to their location on the other side of the screen, the rear cameras cannot be used to recognise the user’s emotions. In turn, a suitable front camera for this task is usually of a much lower quality, as it is most often only used for selfies and video conferences. In recent years, however, this has started to change, and the quality of selfie cameras is beginning to approach that of the rear cameras, with some manufacturers introducing rotating or fully articulating front-and-back cameras. The increase in the quality of the front cameras is very important from the point of view of emotion recognition, as the optical channel is one of the most important and most used in this task.

### 3.2. Microphone

The microphone is a key input device for smartphones, which, in addition to talking, can also be used for many tasks, such as sound recording, eliminating background noise, detecting dangerous sound levels, and voice control in the increasingly popular voice user interface (VUI). Modern smartphones are often equipped with two microphones to improve the quality of the conversation. The voice channel can also be used in the emotion recognition and sentiment analysis tasks. This is possible both on the basis of the analysis of the speech signal as well as on the basis of the analysis of the spoken content after its recognition using the methods of automatic speech recognition (ASR).

### 3.3. Keyboard and Touch Screen

A keyboard and mouse are standard input devices for personal computers with graphical user interfaces and are regularly used by them, especially for tasks involving text input and precise control of a graphical cursor. However, in the case of mobile devices, and especially smartphones, they are of less and less importance. Hardware solutions, such as microjoysticks or a physical keyboard (e.g., in once popular BlackBerry smartphones), have in practice been replaced by touch screens offering direct control and a virtual keyboard. Although, from time to time, there are also models with a physical keyboard or the ability to connect external devices (e.g., through a USB-C connector), these are not popular solutions, especially in the context of currently effective methods of automatic speech recognition and gesture control.

Numerous studies show that the dynamics of the way standard input devices are used depends not only on personal skills but also on momentary emotions or, over longer periods of time, on mood. Therefore, they can be a valuable source of information for emotional recognition algorithms, whether they are physical or virtual.

### 3.4. Depth Sensors

Depth sensors are used for three-dimensional reconstruction of a visible scene by creating an image of its depth. For many years, they have been used in 3D scanners and autonomous robots (SLAM), as well as in game consoles (e.g., Microsoft Kinect), and recently, they have also been used in autonomous vehicles and drones, laptops (e.g., using Intel RealSense technology), and even in smartphones. In the latter, they can be placed both at the back and at the front of the device. In the former case, they usually have a longer range and are used for focusing as well as detecting foreground objects in order to obtain a reliable depth of field and background blur (bokeh) effect. It is also possible to use a rear depth sensor for augmented and mixed reality, 3D photography, and night vision.

In turn, the depth sensors placed on the front of the device have a shorter range and are mainly used to detect the face and create its three-dimensional model. Their basic use is biometric smartphone security (e.g., Apple Face ID). It is also possible to use this sensor for augmented reality, for gesture control, and more entertainingly-for creating animated models of the user’s face (e.g., Apple animoji). Since the front depth sensors are able to locate and track hundreds of landmarks in real time, they can be an extremely valuable source of data for facial expression recognition [36]. Unfortunately, these sensors are still not very popular, and they are found only in the top models of a few manufacturers (e.g., Apple TrueDepth). Many manufacturers, such as Samsung, for example, have postponed their introduction to their products.

Depth sensors can be based on various technologies, such as passive stereo-photogrammetry, which uses two optical cameras, infrared structured light pattern projection, and most popular time-of-flight (ToF) cameras. A variation of the latter technology is Light Detection and Ranging (LiDAR) which generally allows for more accurate and faster depth measurements. A LiDAR sensor is available, among others, in Apple iPhone 12 Pro smartphones. The last three technologies are based on active infrared light emitters and recognise, respectively, the pattern distortion (disparity) caused by the objects’ distance and the time it takes for the light from the emitter to reflect from the object and return to the sensor. ToF cameras are the most popular solution today. Although they do not provide sufficiently high angular resolution and range for effective 3D scanning of large objects and rooms, it is sufficient for the recognition of facial expressions. Since active depth sensors use infrared light, they are better suited to detect unevenly illuminated objects, which is often the case with faces. Unfortunately, this technology is in turn sensitive to direct sunlight illuminating the object.

### 3.5. Accelerometer and Gyroscope

An accelerometer is a sensor that measures linear acceleration along the axis of the smartphone’s local coordinate system. Measurements of acceleration changes allow you to identify the movement of the smartphone in space and even its rotation. Initially, the accelerometer was used to detect screen rotation to change the display mode and to control video games by moving and rotating the smartphone. With the increase in the computing power of smartphones, the accelerometer has also started to be used for electronic stabilisation of the camera and control in virtual and augmented reality applications.

In general, the accelerometer works well for measuring displacement but is less accurate for measuring rotation. For this reason, in mid-range and high-end smartphones it is supplemented with a gyroscope, or gyrometer, that can measure orientation and angular velocity around three axes, e.g., the roll, pitch and yaw of a smartphone. So, the accelerometer knows which way and how fast the phone is moving. It also measures any tremors. The gyroscope, on the other hand, measures deviations from the plane. In practice, both sensors are responsible for similar tasks and work together. Both the accelerometer and gyroscope are used by many applications. Since, when a smartphone is held in hand, its movements and rotations may to some extent reflect the user’s emotional state, the accelerometer and gyroscope can also be used to recognise emotions, although in a multimodal mode as a source of additional information.

### 3.6. Geolocation Sensor, Barometer, and Compass

Most modern smartphones are equipped with at least one geolocation sensor, usually a Global Positioning System (GPS) receiver. GPS calculates the current location of the device from data from GPS satellites, offering users the ability to navigate and track their movement during outdoor activities. These capabilities can be indirectly useful in recognising the user’s activities and emotions.

In order to increase the accuracy of tracking the user’s activity, geolocation is very often supported by additional devices such as a barometer or an electronic compass. A barometer determines the atmospheric pressure and thus the current altitude above sea level. In turn, the magnetic field sensor (magnetometer) measures the strength and direction of Earth’s magnetic fields, allowing the directions of the world to be determined and acting as an electronic compass.

### 3.7. Other Sensors

Mobile devices are equipped with new sensors almost every year. Most of them have very narrow, specialised applications, and some of them are still not very popular or are available only as external devices due to their size, energy demand, or the inability to operate properly in smartphones. The following paragraphs present examples of such sensors.

The *ambient light sensor* consists of photocells that are designed to detect the level of the environmental light’s intensity. This sensor is used to automatically adjust the brightness level of the screen according to changing external light conditions. The *Hall sensor* uses the Hall effect to measure the intensity of the magnetic field. In practice, it can be used to detect the opening and closing of a nonunibody smartphone and its automatic waking-up and sleeping. A *proximity sensor* allows various objects in the immediate vicinity of the device to be detected, even if they are not in direct contact with it. The sensor consists of an infrared LED paired with an infrared light detector and reacts to objects within a few cm. Its main function is to eliminate the phone’s reaction to accidental touch events, such as touching the phone to the ear. It is also used to automatically wake up the screen and turn on the screen and wake the device up or put it to sleep, which saves battery power. A *fingerprint scanner*, or reader, is a biometrics device that recognises persons based on unique physical characteristics of their fingerprints. Although it is still a very popular sensor found in many smartphones, its narrow specialisation limits its use for other purposes.

## 4. The State of the Art

The presented survey has been divided into several sections according to the stages of creating an emotion recognition system. The following sections present the way experiments are organised and the data gathered. Then the methods of extracting and evaluating features from data coming from different smartphone input channels are introduced. Finally, various machine learning approaches applied in the reviewed studies are described and summarised.

### 4.1. Experiment Design

Among the experiments presented in the reviewed papers, two major types may be distinguished. The first type are laboratory experiments performed in controlled environments and usually involving a user in a single session consisting of several stages [37,38,39,40,41,42,43,44,45,46,47,48,49]. The other type is in-the-wild experiments carried in uncontrolled conditions, usually during users’ everyday phone usage. These studies are based on data collected during a period of time, e.g., several days [50], two-three weeks [51,52,53,54,55], a month [56,57,58,59,60], 2–3 months [61,62,63,64,65], or even 2–3 years [66,67]. Some researchers take on both types to evaluate their methods [68,69].

One of the parameters influencing the credibility of the obtained results is the number of experiment participants. Usually, they represent a particular group of participants, e.g., students. However, it is also possible to engage a wider spectrum of the population. The number of participants in the reviewed studies varies from one to thousands in several cases, with a median value of 23. The exact numbers are given in Table 2.

In the case of laboratory experiments, the emotional states are usually induced. A simple method for this is presenting videos [40,68] or pictures [45,70] selected according to the desired emotions. Another method is based on experiencing some memories relevant to the affect [42,48]. Conversation is another activity able to elicit emotional states. In [49], exciting, shocking, rude, and confusing conversations were carried on to induce different emotions. In [43], negative emotions were induced while chatting with a bot and commenting a previously seen video, which was supposed to simulate typical social media tasks. Various methods were used to bring about stress, e.g., time limits given for a task [38,56,69], sounds and vibrations [56,69], and unexpected behaviours of the device [56]. It is also possible to design a game which makes the player always lose or win [44] or has a dull and a fascinating mode which is designed to induce negative and positive states, respectively [46]. The experiment protocol usually consists of several stages ending with self-reports with a calming stage between them, which may incorporate relaxation music [69] or video [42,43,49].

### 4.2. Data Collection

Depending on the sensors applied, continuous sensing may lead to privacy or phone energy consumption problems [71]. Therefore, data are usually recorded periodically or depending on a context. Moreover, assigning reliable labels to the captured data samples also brings about some difficulties. The following two subsections present the solutions applied to make the collection of training data feasible.

#### 4.2.1. Limitations

Smartphones are very convenient for the user; however, due to their small size, they have various limitations that should be taken into account when developing and using mobile applications. One of the issues is the operating time due to the limited battery capacity. The basic idea to cope with this problem is to capture data periodically.

In [65], for example, information on location is collected every hour, in [51] every minute, in [63], the sampling rate for the accelerometer is 5 Hz. In [72], data collection lasts for 1 h, but sensor data are gathered every 10 min and each collection lasts 5 min. In [60], the data collection interval is set to 5 min and the sensors are scanned for 15 s. In [73], accelerometer data collection starts when a movement is detected and the phone is not being used, then the recording lasts for 20 s. In [74], sensor and typing data are collected only when the user is typing. Sometimes the energy saving comes at a cost of lower accuracy. In [66], for example, coarse-grained GPS data are collected, which is easier and quicker to sample and more energy-efficient but may be less accurate. In [75], the movement precision is set to 10 m and the location upload is performed once every minute. The frequency of Bluetooth scanning may be also reduced to keep from draining the battery, e.g., to every 5 min [59,76].

Not only recording but also transferring data is especially energy-consuming. A common approach to maximise the phone operating time by saving battery is to store the recorded data on the phone and send it to a server once a day when a WiFi connection is available [58,60,62,66,67,77]. Network load may also be a limitation. One of the solutions is sending data to server nightly [57,61]. The models are trained in the cloud and then delivered back to the smartphone [61].

Another problem may be insufficient local memory. This may force the need to transfer data or only the extracted features to the cloud. For the sensor data such as microphone sound and light, the authors of [60] compute some measurements and submit the extracted features to the server without keeping the original data. In [58], the accelerometer is used continuously, but it records a new value only if it differs from the previous one by at least 0.5 m/s${}^{2}$ to reduce the amount of data stored.

The amounts of training data together with the computational complexity of model training algorithms make the training on a phone infeasible [78]. Therefore, the models are usually trained on a server and then sent back to the phone, where they may be used for prediction [60,61,62,66,67,72,77,79,80]. In [58], not only the training but also the inference is performed on the server and only the daily mood is sent back to the user.

#### 4.2.2. Data Labelling

Labelling data samples collected from different sensors, sometimes at different moments and not necessarily at the moment of filling in a self-report, requires putting a lot of effort to get reliable class labels.

In the case of in-the-wild experiments, experience sampling method (ESM) is a common tool applied to label data samples read from phone sensors. ESM is a tool used for sampling people’s experiences in daily life [81]. It is achieved by filling in a repeated survey, which may be completed in response to an event or at specified times. There are several limitations of experience sampling methods. They can be time and resource-intense and potentially burdensome to participants. Therefore, the design of a sampling protocol has to take into account maximising reliability of the collected data while minimising participants’ burden.

Usually, one of two typical schedules are applied to select the moment to ask a user for a self-report, i.e., time-based and event-based. A time-based schedule is most common. It may be performed several times a day [52,60,61,64,69,75,82,83], sometimes more often, e.g., 12 times a day [50] or every hour [84]. Usually some interval between subsequent reports is required [60,83]. Sometimes users have more freedom in selecting the right moment and only acquire notifications or are able to initiate self-reports themselves [51,58,61,67]. Another idea is to apply an event-based schedule, e.g., when a user switches an application [78,79,85], after the end of a game level [86], after detecting a pause in typing [85], after performing sufficient amounts of typing and a minimum time elapsed since the last ESM [87,88].

A study presented in [54] focuses on comparing the effectiveness of three types of ESM. Trigger ESM asks a user whenever her behaviour changes, which is detected using an activity recognition API. Unlocking ESM is accomplished when a user unlocks the screen. The last one is randomised ESM. If a number of reports are obtained during a predefined period, which results in one feature vector, the label is assigned according to the most frequently answered self-reported emotion. Applying the unlocking ESM leads to models with the highest accuracy in most cases.

Although a study presented in [89] does not concern smartphones, its conclusion refers to any interaction in general. Significant differences have been shown between emotional responses collected at the end of an interaction and during the interaction. Therefore, it is essential to assign the right moment to ask the user about her emotions. Deliberate ESM design may lead to better quality of user responses, which in turn may improve the classification accuracy of the trained models.

A proposition of an ESM schedule focusing on a balance between user fatigue and the timeliness and accuracy of responses has been proposed in [90]. In this Low-Interference High-Fidelity (LIHF) ESM schedule, when an event is detected, a survey appears only if a predefined time since the last one has passed. All of the events between two successive surveys are labelled with the input provided by the user. In their further study presented in [91], the authors also trained a machine learning model to detect the inopportune moments for self-reports. To get the examples of inopportune moments necessary to train the model, information from reports was used, as it allowed the users to select a NoResponse option. The proposed methods lead to reducing the probing rate and collecting the self-reports in a more timely fashion. As a result, more valid labels were obtained and an improvement in classification accuracy was achieved.

In the case of laboratory experiments, a self-report is presented to a participant at specified points of the session, usually at the beginning, end, and after each step or task if a session consists of several of them [38,42,68,69]. Sometimes the labels are assigned according to the emotions elicited, assuming the induction was successful [44,46].

A survey presented to a user may have various forms. The simplest one is to provide a kind of option button for each analysed emotional state, which enables a binary representation for that emotion [49,88]. Another way is to report the emotional state on a Likert scale [92], which may be applied for any emotional state [42,50,52,53,64,67,68,69,76,93]. In the case of pleasure, arousal, and dominance, Self-Assessment Manikin (SAM) [94], which is a pictorial version of the scale for the PAD dimensions, is often used [38,43,49,68,84].

Some studies assume a user may experience mixed emotions and let the user select more than one state, e.g., in [65] the user might select two emotions and the second one is treated as an additional feature, and in [60], where compound emotions are considered and represented as a vector of scores given to six basic emotions.

Other scales, designed for specific emotional states or moods, are also sometimes applied. One of them is Positive and Negative Affect Schedule (PANAS) [95], which is a questionnaire consisting of two 10-item mood scales to measure positive and negative affect, and was used, for example, in [38,43]. Photographic affect meter (PAM) [96] is another tool to express one’s positive or negative affect by selecting a picture that best fits the mood, and it was applied, for example, in an application designed to assess the well-being of students [62]. Another one is Perceived Stress Scale (PSS) [97], consisting of 14 questions, which can be applied to label samples for a stress detection task [52]. Depression, Anxiety and Stress Scales (DASS) [98] is a tool consisting of 42 questions giving a score for each of the three states. It was used, for example, in [99] to detect depression, anxiety, and stress through touch dynamics.

The number of levels available at the moment of filling the self-report does not have to correspond to the number of predefined classes to be recognised later. It often happens that selected levels are scarce in the collected data set and there is a need to merge the levels, reducing their number [43,53,73]. During this process, individual characteristics may be taken into account, as in [53], where, due to the fact that people have different predisposition to boredom, personalised z-score normalisation was applied to assign labels while transforming from a 5-point scale to binary.

A survey may refer not only to emotional states but also to other information, e.g., the number of hours of sleep [59], physical activity, location, or social interactions [66,67].

Some researchers suggest that insight into past behaviour makes users operate more efficiently [100]. The participants feel more engaged with the study, which is manifested in terms of improvement in participation duration and the number of self-reports collected during the study.

### 4.3. Data Representation

Raw data recorded via different sensors are processed to extract features describing users’ behaviour. Most methods are based on hand-crafted features. Some algorithms accept input data in their original form, which is usually time series. The following subsections present various parameters calculated on the basis of data from different sources, which have been proposed and tested in the reviewed studies as indicators of human emotions.

#### 4.3.1. Touch Dynamics

The primary source of input data in the case of a smartphone is the touchscreen, which enables performing various gestures, such as tapping, scrolling, swiping, etc. The way a user performs gestures depends on the user, which is often applied in user authentication [101,102,103]. Moreover, numerous factors, e.g., emotional state, device type, screen size, may affect gesture dynamics and shape even when they are performed by the same user.

One of the main functions fulfilled with the use of a touchscreen is the virtual keyboard. Keystroke dynamics has been widely investigated in the area of emotion recognition of computer users [104,105,106]. Similar keystroke parameters, describing timing and frequency characteristics, are also applied in the case of smartphones.

The first set of keystroke parameters are timing characteristics. Among them, the equivalent of hardware keyboard flight time is one of the most common. This feature represents the time between two consecutive tap events while typing [43,55,69,74,78,79,85,87,88,107] and is sometimes called the intertap duration. The value of this parameter is usually averaged over all taps performed during a typing session or a time window. Ghosh, in his numerous studies, also proposes calculating similar parameters taking into account a major group of sessions identified after clustering them into two groups on the basis of their length [55,78,87,88] or after rejecting outlier values [79]. He also incorporates the number of intertap duration values greater than 30 s as a feature [79]. Another parameter taken directly from keyboard data analysis is tap duration, sometimes called hold time, which is defined as the time a finger touches a screen during a single touch [43,69,107]. Two other features provide some information on the speed between touch events—the down-down and up-down speed—which are the times between two consecutive touch downs and between a touch up and the subsequent touch down, respectively, and they are normalised by the distance [49]. Other common typing characteristics are typing speed [38,43,46,100,108], typing time [51,79,108], and session duration [55,69,78,79,87,88,100,107]. Typing time per character or per word may also be taken into account [79].

The second group of keystroke parameters are frequency characteristics, which describe how often selected keys are touched. They usually measure the frequency of using backspace or delete [38,46,51,55,69,74,78,79,87,88,107,108], enter [51], and the space bar [43]. The use of special symbols is also worth taking into account [51,55,78,79,87,88] or the use of keys designed for some activities often performed while typing, such as, for example, *send* or *change language* [43]. Various touch counters are also considered, i.e., the number of touches [51,108] or the number of touches outside of the keyboard layout [43]. Finally, the simple text length [55,78,87,88] or number of letters [74] may also add valuable information.

There are several features which may be a source of information on mistakes made while typing, which may be correlated with emotional states. Apart from the already mentioned backspace and delete usage, it is also worth noting the percentage of incorrectly typed words [69,107], the length of erased text [51], or the maximum number of characters typed without pressing delete for a predefined time [46].

In addition to the presented parameters, which may also be implemented for hardware keyboard users, touchscreens provide additional possibilities, which may also be considered when designing keystroke typing features. It is possible to measure the pressure and size of a touch [38,43,69,107]. These parameters might be good indicators of arousal level. Moreover, it is also possible to measure a tap movement which usually occurs, even in the case of a single touch [43,69,107].

The parameters are usually mean values calculated on the basis of all touches in a session or a time window [43]. However, some studies include other characteristics, such as standard deviation, variance, minimum, maximum, range, median, or other percentile values [43,68,69,79], which significantly enlarges the length of the feature vectors.

In contrast to representing a session by a single feature vector, it is also possible to describe it as a sequence of vectors representing subsequent taps by the following parameters: intertap duration, alphanumeric (1/0), special character (1/0), backspace (1/0), touch pressure, touch speed, and touch time [109]. Another form of sequential input has been proposed in [49], where the sequence consists of three types of heat maps, each created on the basis of 180 s windows with a 5 s shift. The maps contain pressure, down-down speed, and up-down speed, respectively. Obviously, such a sequential representation entails applying suitable knowledge representation which is mentioned in Section 4.5.

Apart from typing performed with touch gestures, a touchscreen enables entering more complex gestures, e.g., scroll or swipe and any other strokes. These data are also processed to extract features, which may be useful for emotion recognition. Feature values are calculated on the basis of single strokes [99] or may be averaged over a series of them from a predefined time window [44] or session or a predefined number of consecutive strokes [41].

The tap is the simplest gesture and most of the typing features apply to any tap as well. These are: size [69], pressure [37,44,45,68,69], duration [45], time between two subsequent touches [45], and touch movement [68,69]. Sometimes, the numbers of touch events are also taken into account separately for finger down, up, and move if any movement occurs during a touch [44]. An original touch parameter, i.e., the relation between the touches on active and passive areas, was proposed in [37]. Mottelson also proposes tap characteristics that provide some information on touch precision, i.e., the distance between the tap and the target centre [68]. Moreover, he takes into account the angle between the horizontal line intersecting the centroid and the line connecting the centroid and the tap and a similar angle but measured relative to the previous interaction.

More complex gestures, such as swipe or scroll, can be described by a set of simple parameters, e.g., stroke length [41,69,86,99], distance between the beginning and ending point of a stroke [69,86], speed [41,68,69,86,99], gesture duration [41,69,99], distance of the gesture from the centre of the screen or from the selected corner [69], pressure [41,69,86,99], and touch area [99]. In the case of pressure and touch area, the feature value has to be estimated on the basis of all points constituting a stroke, for example by averaging. In the case of pressure, its decline, defined as the difference between the pressure value at the starting and ending point of a gesture, has also been proposed [68].

It is also worth measuring the linearity of a stroke. The easiest way is to take into account the relation between the stroke length and the distance between its starting and ending points [99]. Another idea is to measure the average distance to a predefined line, when a user is supposed to follow it [68]. For strokes, which are supposed to be horizontal or vertical, the estimation of linearity may be simplified by calculating the horizontal or vertical distance between the first and the last point of the gesture [69]. In the mentioned study, another linearity parameter was defined as the sum of the above distances between all pairs of consecutive points. Another way is to incorporate the variance of angle between points and horizontal or vertical line [99].

Balducci also introduced a set of features calculated by taking into account all pairs of consecutive points belonging to a swipe or all pairs between the starting/ending point of a swipe and any other [99]. These parameters are extracted separately for eight predefined directions, and they include the percentage of touches in each direction and the variance of the direction of the vector determined by the mentioned pairs of points.

Special features are designed if a gesture involves more than one finger. For example, in the experiment presented in [68], two-finger gestures performed for scaling are represented by the distance between the fingers, the difference in angle between the fingers and the centroid at the beginning and end of the interaction, and the average distance to the target scaling.

If the application used to collect gesture data is going to be used on different mobile phones with various screen resolutions, some of the features related to distances should be normalised by taking into account the width and height of the screen [69].

#### 4.3.2. Movements

An accelerometer is usually applied to estimate the movements of a phone. It returns its measurements along three axes, thus at time $t_{i}$, three values are read from the sensor: $x_{i}$, $y_{i}$, and $z_{i}$. The aggregated acceleration value at time $t_{i}$ is defined as follows:

(1) $$a_{i}=\sqrt{x_{i}^{2}+y_{i}^{2}+z_{i}^{2}}.$$

The first step in processing the obtained data series is often noise reduction. This is usually performed by applying a moving average filter to each of the three axes [40,48,73]. The size of the moving window used for noise reduction, which is usually 3 or 5, may affect the final effectiveness of the trained models [40]. A sequence of the acceleration values read during a time window are used to extract features. The parameter values may be calculated either on the aggregated series [110] or only on individual of the three axes [40,43,60,82] or by applying both approaches [48,63,68]. In any of these cases, the feature vectors are extracted on the basis of segmented data series, which results in obtaining one feature vector for each segment. The common way to split the series into segments is a sliding window, which may be either overlapping or not, and may be of different length specified by a time period, e.g., 5 s [43,74,82], or a number of samples, e.g., 128 [40]. If the sensor data is recorded for a specified period, e.g., 5 min, then it leads to a number of feature vectors being extracted. Usually, all of these vectors are added to the data sets used for further processing. Another approach is to calculate some statistics for each feature, e.g., mean, maximum, minimum, and create a single vector for the whole period, as was done in [63], where the final feature vector represents a 2-h period. Another idea is to identify the segments taking into account the waveform shape. In [73], for example, acceleration data are processed to identify walking segments, and features are extracted on the basis of periods of motion, which contain at least four steps. A similar solution was implemented in [48], where after identifying the steps, stride segmentation was proposed. In this case, one segment contains two successive placements of the same foot. In contrast to the sliding window, these types of segmentation may result in segments of different lengths. It is also possible to extract a feature vector representing the whole session, e.g., in [68], features were calculated on the basis of a predefined period of a game played by a user.

Features extracted from the acceleration time series may be divided into several subsets. For the readability of the section, we split them into three groups. Low-level parameters are calculated directly based on acceleration time series, e.g., descriptive statistics. Mid-level features require more complex processing, e.g., parameters calculated based on FFT coefficients. High-level features are obtained by further processing, sometimes using machine learning methods, and they represent information contained in data in a more meaningful form, e.g., activity type. It should be noted that the division presented here is not the only possible one and that the bounds are not strict, especially between the first two groups. One might assign the same feature to another group.

##### Low-Level Features

The basic group of low-level features often extracted on the basis of accelerometer data series are descriptive statistics, i.e., mean, standard deviation, variance, mean absolute deviation, minimum and maximum value, index of the minimum and maximum value, range, median, interquartile range, etc. The other group are parameters known from signal processing, e.g., root mean square, energy, power, magnitude, signal magnitude area. These features are presented in Table 1 together with references to studies, where they were implemented. Most of them may be calculated separately for each axis.

##### Mid-Level Features

This group of features contains a number of parameters characterising the distribution shape. Apart from the already mentioned skewness and kurtosis, which belong to descriptive statistics and measure the asymmetry and the heaviness of the distribution tails respectively, the waveform shape may be also described by identifying crests and troughs, which are local maxima and minima. Several features may be calculated on the basis of them, e.g., crest mean, trough mean, and the maximum and minimum difference between the crest and trough [82]. In [48], the zero crossing rate and slope sign change were also incorporated.

Another characteristic worth investigating is jerk. It is defined as the first derivative of acceleration at time $t_{i}$, and it can be calculated as follows:

(2) $$\begin{array}{c} j_{i}=\sqrt{j_{x,i}^{2}+j_{y,i}^{2}+j_{z,i}^{2}} \end{array}$$

(3) $$\begin{array}{c} where\;j_{x,i}=\frac{x_{i+1}-x_{i}}{\Delta t},\;j_{y,i}=\frac{y_{i+1}-y_{i}}{\Delta t},\;j_{z,i}=\frac{z_{i+1}-z_{i}}{\Delta t}. \end{array}$$

The mean and standard deviation of jerks are features which may indicate sudden movements [73,110].

Apart from time domain features, there are also numerous parameters from the frequency domain. First of all, it is possible to apply Fourier transform on the acceleration data series and use a predefined number of FFT coefficients as feature values [40,48,63]. The coefficients are also used to calculate the mean or maximum [48]. Moreover, it is also possible to extract energy as the sum of the squared discrete FFT component magnitudes of the signal [48,63]. Some studies include the peak magnitude, which is the maximum value of the magnitude, and peak magnitude frequency, which is the frequency of the maximum magnitude, peak power, and peak power frequency [63]. These features might be useful in activity recognition, because it has been shown that different types of activities have different energy distributions over the frequency spectrum [111]. Entropy over the power spectrum is also sometimes used as a feature [63]. Finally, a number of experiments incorporate power spectral density (PSD), which is a measure of the frequency distribution of power of the time series [40,48,73,110]. The mean of the PSD is the size of the average power per unit of bandwidth. The standard deviation of the PSD shows the degree of dispersion in terms of power [40].

Another transform providing more time-domain characteristics is wavelet transform. In [48], each series of data was decomposed into five levels using a Daubechies 2 wavelet mother. The obtained coefficients were used to extract two types of features: the sum of squares of coefficients and the sum of absolute values.

##### High-Level Features

One of the features we put into this group measures deviation from a user’s usual behaviour. It was proposed in [67] as the Dynamic Time Warping (DTW) distance between the accelerometer series from the observation interval and the average readings of the same user obtained during the same interval on other days. The feature value indicates how the acceleration deviates from the user’s usual behaviour.

Numerous studies include a parameter describing the device shaking, which may be implemented on the basis of acceleration changes. This feature value can be calculated as changes in aggregated acceleration [68] or by identifying the device’s position change above a predefined threshold [46,51,108,112]. In [112], the shaking time and severity of shaking were also taken into account.

A commonly used feature, which may be extracted on the basis of accelerometer data, is the activity type or even the intensity of activity. A simple approach was proposed in [72], where three states, i.e., run, walk, and silence, were identified by comparing the aggregated acceleration with predefined threshold values.

An example of such a feature was also implemented in [64], where, on the basis of acceleration variance, each segment was assigned a value of high, low, or none. The physical activity level was then represented by the percentage of high activity segments during a given period. The proportions of sitting, walking, standing and running states were also incorporated in [58]. In [82], the rich set of features previously mentioned was enlarged by three more features indicating steady, slow and fast periods [82].

In [84], raw *x*, *y*, and *z* values were used to classify data using an SVM classifier either to a moving or idle class. The result of the classification was then used as a feature value. A classifier for activity recognition was also applied in [77,113], where a data sample was represented by a set of time and frequency domain features, and then a naive Bayes classifier was used to assign a data sample to one of the following classes: driving, stationary, running, walking. In [62], a decision tree was trained to classify accelerometer feature vectors into five classes: stationary, walking, running, driving, cycling. A high accuracy of 94% was achieved in this case. Moreover, the duration of daily activity was estimated as the sum of all active periods, where a 10 min period was recognised as active if the ratio of the nonstationary state exceeded a predefined threshold. In this study, the activity was also used as additional information to infer sleep duration, beside light information, phone usage, and sounds.

Due to the availability of the Google Activity Recognition API, the information on activity type for Android phones is often received from this service [50,52,93]. It recognises several states: in vehicle, on bicycle, on foot, running, still, tilting, walking, unknown. A similar service may be used for iOS users, i.e., the Apple CMMotionActivity API providing stationary, walking, running, automotive, cycling, and unknown states [93].

Some studies take into account more detailed information on movement by detecting steps on the basis of acceleration time series. They extract the step duration [48] or step count [60]. In [73], where emotions are recognised only while walking, more sophisticated step features were designed, i.e., mean peak acceleration, which is the average of the greatest values from the steps in the window, standard deviation of the mean peak acceleration, and mean step duration. Moreover, some of the parameters mentioned before, e.g., mean acceleration, mean jerk, skewness, and kurtosis, can be calculated separately for each step, and the final feature values would be averaged over the steps. It may lead to slightly better performance in some cases [110].

Most features presented for an accelerometer can be also implemented for a gyroscope or magnetometer. In [82], the same features previously described were extracted for an accelerometer, gyroscope, and magnetometer. The set includes maximum, minimum, mean, standard deviation, wave number, crest mean, trough mean, and the maximum and minimum difference between the crest and trough. Each of the parameters was calculated for each of the three axes of the three sensors. The same idea was implemented in [48], where all accelerometer parameters, except for step length and step duration, estimated only on the basis of *x* series, were implemented for gyroscope data. In [60], the mean and variance of *x*, *y*, *z* for gyroscope and magnetometer values were extracted. In [43], the minimum, maximum, mean, and standard deviation were calculated not only for the raw *x*, *y*, *z* gyroscope series but also for *pitch*, *roll*, and *yaw* values. In experiments presented in [68], some statistics were calculated on the basis of raw values and also changes in aggregated rotation were taken into account. In [80,112], rotation time and average angular velocity were extracted.

#### 4.3.3. Location

A GPS sensor provides information on the phone’s location in the form of the latitude and longitude coordinates of a geographic location. These two parameters are often incorporated in a feature vector, either directly [60,108] or after some processing as is described below.

Reading GPS for a period of time with a predefined interval may lead to a large number of different locations obtained, which is not necessarily readable and difficult to analyse. Therefore, a common approach is to limit the possible locations to a predefined set. The easiest approach to perform this task is to involve the user in labelling her locations. In [66,67], users were periodically asked to choose one of the following places: *home*, *work*, *family/friend’s house*, *restaurant/cafe/pub*, *in transit*, *other*. When a self-report on emotions appeared, the location sensed temporarily closest to the report was found and a corresponding place assigned. The place label may be treated as a feature value. Home and office locations were also manually entered in [84].

In contrast to manual tagging, automatic clustering of all recorded locations is often performed using either the k-means algorithm [56,58,70,82,83] or DBSCAN [57,61,64,72], which in contrast to k-means, does not require a predefined number of clusters.

Some additional constraints are sometimes imposed for the clustering, e.g., maximum diameter of a cluster [56], maximum distance between clusters [83], time spent at a location longer than a predefined amount [70], or stationary locations, which are first identified on the basis of movement speed compared with a threshold [83].

The obtained clusters can be then assigned meaningful labels. In [93], for example, the labels, i.e., *home*, *store*, *leisure*, *work*, *restaurant*, *health*, *gym*, *other house*, *religious*, are assigned on the basis of the OpenStreetMap geodatabase.

There are various features which may be extracted on the basis of clustered locations, and their usability depends on the application. The simplest approach is to use cluster label or ID as a feature value [56,58,70] or the number of visited locations [82]. In [57,61], the features represent the number of visits in the top ten most visited clusters during a 3 day period. Another idea is to incorporate the percentage of time spent at each type of location during a time window [93] or the entropy of time spent in different locations [82,83] defined as:

(4) $$Entropy=-\sum_{i=1}^{N}p_{i}\log p_{i},$$

where $p_{i}$ is the ratio of time spent in cluster *i*, and *N* is the number of clusters.

Processing raw time series obtained from periodical GPS readings also leads to several interesting features. It is possible to calculate average values and the standard deviation of latitude and longitude over a predefined time [75]. The coordinates also enable distance parameters to be extracted, e.g., the average distance from work or home [75]. In [62], for example, outdoor mobility, defined as the total travelled distance per day, was calculated.

Several interesting features were implemented in [83]. The authors define transition time as the percentage of time spent while moving during the day and total distance as the total movement distance in a day. Moreover, they apply location variance being a measure of the movement scope:

(5) $$LocationVariance=\log(\sigma_{lat}^{2}+\sigma_{long}^{2}).$$

The study also takes into account how regular the patterns of the user’s movement locations are. This parameter is called circadian movement and is defined as follows:

(6) $$CircadianMovement=\log(E_{lat}+E_{long}),$$

(7) $$E=\sum_{i=1}^{N}psd\left(f_{i}\right)/\left(i_{i}-i_{N}\right),$$

where *f* is a bin in the frequency domain analysed from GPS locations by least-squares spectral analysis, *N* is the number of frequency bins corresponding to 24-h periods, *i* is the index of the frequency bin, and $psd(f_{i})$ is the power spectral density at frequency bin $f_{i}$.

GPS enables outdoor locations to be inferred. In the case of indoor environments, it is possible to take advantage of other channels, i.e., Bluetooth or WiFi [60,62,114]. For example, in [60], access point identifiers are scanned every 5 min and the frequencies of the most often occurring points of a user become feature values. This indicates the indoor locations a user visits often. Some studies analyse WiFi scan logs to estimate the distance a user travels inside particular buildings during a day as the total indoor mobility [62]. In [53], the feature value indicates which WiFi network the phone is connected to, i.e., *home*, *work*, *other*, *unknown*.

As in the case of GPS locations, WiFi access points can also be clustered. For example, in [64], they were clustered with DBSCAN and the analysis focused on location changes by comparing location counts in two consecutive hours.

Indoor locations were also identified on the basis of Bluetooth. In [114] the number of other devices in the proximity was used to identify *workplace* for example.

#### 4.3.4. Social Interactions

Bluetooth is usually used to monitor social interactions by scanning other Bluetooth devices in close proximity. The presence of the devices may indicate the intensity of contacts with others, which may have something in common with mood. One of the parameters extracted from information provided by Bluetooth is the number of devices seen or the number of connections [56,59,62,72,76,82,115,116] over a predefined period. In [59], both daily and overall, i.e., over a month, sociability is taken into account.

It is also possible to incorporate time spent with others [56], maximum time that a Bluetooth device is seen [72], and the identifiers of devices seen for more than a specified amount of time [72].

In [76,115,116], the diversity and regularity of Bluetooth contacts was introduced. To represent diversity, three features were added, i.e., the entropy of proximity contacts, the ratio of unique contacts to interactions, and the number of unique contacts. Regularity was described by the mean and variance of the time intervals for which a device was seen. Moreover, for each basic feature, several second order features were extracted on the basis of data collected in a time window, i.e., mean, median, minimum, maximum, selected quantiles, variance, and standard deviation. The same set of features was also calculated for 2- and 3-day backward-moving windows. This was performed in order to take into account the fact that past events might also influence the current emotional state.

#### 4.3.5. Ambient Light

A light sensor provides us with some information about the environment. It measures the amount of ambient light and outputs the result in SI lux units. The output value may be used to infer the phone’s current status. The values significantly differ depending on the location of the phone. They are low when the phone is in a bag, for example, higher under normal light, and highest outdoors [112].

Therefore, it is possible to extract some features describing phone usage. The illuminance of ambient light may be directly treated as a feature value [51,53] or it may be discretised to several possible levels [50]. If the amount of light is sensed periodically, statistics such as mean or variance may be extracted [60]. More interesting features are based on the distinction between dark and bright measurements. It is then possible to extract the proportions of dark, bright, or dark-to-bright measurements [52,60]. Other parameters include the proportion of time a phone was not used, was used indoors, and was used outdoors [82]. The light sensor can also be incorporated in predicting bedtime, together with other parameters describing phone usage, activity, and sound [62], which often correlates with the emotional state.

#### 4.3.6. Additional Information

Although this review focuses on recognising emotions on the basis of data from the selected set of input channels presented in the previous subsections, it has to be noted that most studies utilise other information as well, both from other hardware sensors and from other sources such as the environment context or phone usage.

Apart from the sensors considered in our survey, some papers also use the sensors excluded from this review. The microphone is often used [50,52,53,54,60,62,64,67,77,112,113], usually in order to detect the noise level or speech periods.

Among other than hardware sources, numerous reviewed studies also incorporate information on phone usage patterns [50,52,53,54,57,58,61,64,65,67,69,70,72,76,82,84,114,115,116]. This type of information may be extracted mainly from calls, messages, and the types and frequency of applications used.

Some information on users’ activity can also be concluded from the battery state [114], screen on/off states [84], or the phone calendar [50,57].

Context information is also valuable, because it affects behavioural patterns, e.g., time or day of the week [51,65,75,84] or the weather [51,54,76,114,115,116].

The use of emoticons in messages is straightforward information on emotions, so it is also worth taking into account [72,100].

### 4.4. Feature Evaluation

Due to the high number of extracted parameters, feature selection is usually performed to reduce the complexity and to remove irrelevant information. The usability of particular features, measured as their predictive power, depends on the given task or application and the given set of data. The results of some studies indicate that different subsets of features are good in identifying different emotional states [99]. Moreover, optimal subsets also vary between users [56,57,60,61]. Therefore, it is difficult to indicate a set of universal features. However, some insights are worth emphasising.

To find an optimal subset of features, it is necessary to choose a strategy to search the space of possible feature subsets and a criterion to evaluate the subsets in order to select the optimal one. The performed survey shows that two popular search strategies are usually applied. The first one is to evaluate each single feature and choose the best ones in the sense of a criterion used. The disadvantage of this approach is that it ignores possible dependencies among features. The popularity of the method arises from its simplicity. The other strategy often applied is sequential selection, which may be either forward selection, where features are added to the subset, or recursive elimination, where features are sequentially removed. The sequential methods take into account feature correlations to some degree. To evaluate a subset, a filtering or a wrapper method may be performed. In the case of filtering methods, a feature subset is evaluated on the basis of a selected criterion, whereas in the case of wrapper methods, the subset is evaluated on the basis of the results of a trained model [117].

Both search strategies and evaluation approaches are often applied among the reviewed papers. Recursive feature elimination was performed in [50,99,110] and sequential forward selection in [61,63]. They are usually accompanied by a wrapper approach to evaluate a feature subset, e.g., linear SVM [99] or linear regression [61]. The filtering approach to feature selection is also popular, e.g., using information gain criterion [55,69,87,88], Gini coefficient [76,115] or ReliefF [60].

Numerous parameters from smartphone sensors have turned out to have discriminative power in recognising the levels of pleasure and arousal. An experiment described in [49] shows that touch pressure values recorded while typing might be good predictors of arousal, whereas down-down and up-down speed are better for valence. The results presented in [38] suggest that under negative emotions, typing speed decreases and the error rate increases. Another study focusing on features representing touchscreen strokes shows that stroke speed and pressure under negative emotions are significantly higher than under positive and neutral states. Moreover, the stroke time is longer and the length is shorter under positive and neutral emotions [41]. Discrimination between low (bored, relaxed) and high (excited) arousal can also be performed on the basis of the speed and distance between the beginning and ending point of a stroke [86]. Pressure and length turned out to be suitable along the valence dimension, i.e., pressure at discriminating frustration, length features at discriminating bored vs. relaxed [86]. In [68], it was reported that positive states cause slower and more accurate motor behaviour.

Accelerometer data collected while moving, but not using the phone, lead to several good predictors of arousal and pleasure. In the case of arousal, these are: mean acceleration, standard deviation of acceleration, standard deviation of mean peak acceleration, mean jerk, mean step duration, skewness, and kurtosis. Whereas for pleasure, these are: standard deviation of power spectral density [110]. Some of these findings are in compliance with the observation that activity, which may be identified by accelerometer data, is one of the determinants of happiness [3]. In the experiments presented in [67], data from the accelerometer and microphone (noise) turned out to be more informative of a user’s mood than their sociability parameters such as calls and messages. It was also reported that positive emotions lead to bigger movements and fewer changes of orientation of the devices while using it [68].

Location features also demonstrate high discriminative power in this area, e.g., pleasure turned out to be positively correlated with the number of visits to selected locations [61]. Negative valence was associated with significantly higher location variance, number of clusters, and entropy [83]. Extreme arousal levels, both high and low, are associated with higher location variance and transition time, which means more frequent movement as compared to the neutral state. In the case of high arousal, the patterns of movement locations, measured with circadian movement (6), are more regular [83].

Stress is one of the affective states often investigated. Therefore, much effort has been put into identifying stress detectors. Among touchscreen characteristics, an increase in typing pressure is usually correlated with stress [42]. Some features describing intensity of touch, i.e., minimum and maximum, were also identified as good stress predictors in [37]. The higher the stress level, the more significant the relation that was observed.

Scroll and swipe gestures can also be used for stress inference. In [69], it was shown that under stress, scroll gestures were faster and the strokes were smaller, whereas swipe gestures showed lower duration and grater contact area. Stroke features calculated on the basis of sequences of point pairs, mentioned in Section 4.3.1, turned out to be good predictors in recognising the levels of stress, anxiety, and depression [99].

Stress may also affect tapping. While performing a task which requires tapping circular targets, the tapping was less accurate under stress [47]. This corresponds to one of the mentioned observations: that a positive state leads to more accurate motor behaviour [68].

An experiment presented in [63] revealed a set of the best accelerometer features for stress recognition. Although feature selection was performed individually for each user, some features were selected more often. The top five are: magnitude, standard deviation of *x*, *y*, *z*, minimum energy, maximum value of *x*, *y*, *z*, and peak magnitude frequency. A study described in [37] also revealed a strong relationship between stress and acceleration.

Location parameters can also be used to recognise stress. It is possible to infer about stress on the basis of diversity in colocation interactions [76]. This complies with an earlier remark that negative valence is associated with higher location variance, number of cluster, and entropy [83].

Several interesting conclusions were drawn on the basis of a correlation analysis between sensor data from smartphones and the mental well-being of students [62]. The study revealed a correlation between stress and sleep duration estimated on the basis of light sensor and other information, i.e., students sleeping more experienced less stress. Moreover, students that were more social and around people, which was measured using Bluetooth, were more flourishing. Similar findings on the correlation between mood and sleep duration were presented in [59]. They also show that people who are often in a negative mood demonstrate significantly lower sociability. In [76], features describing social proximity turned out to be good indicators of happiness. In [60], where compound emotions were recognised, several sensor features, together with some others such as, for example, phone usage, were selected as good discriminants for most users, i.e., parameters from light, accelerometer, and audio. Among the features gathered while typing messages, typing speed, text length, shaking of the device, and location turned out to be good for recognising happiness, surprise, anger, disgust, sadness, fear, and neutral state [51].

### 4.5. Model Training

Most methods applied to recognise the emotional states of smartphone users fall into the category of supervised learning. The models are trained on the basis of sets of feature vectors accompanied by target classes, which indicate emotional states. Among these methods, both classification and regression models are applied. All popular models have been implemented and tested in the task of emotion recognition based on data from smartphone sensors, i.e., random forests, which seem to be the most common [41,45,48,54,55,65,76,78,82,87,88,99,115,116], decision trees [37,39,63,64,69,74,118], SVM [40,43,45,48,68,69,72,73,86], discriminant analysis [86], naive Bayes [45,50,63,108], Bayesian networks [51,84], factor graphs [58,60,70], multilayer perceptron [73], deep neural networks [49,67,79,109], generalised boosted model [76], linear regression [61,74], and logistic regression [45].

Although classification models are the most common in this application area, there are some research studies where regression models are also trained [61,68,75]. In [75], regression on a continuous scale produced better results than binary classifiers applied for predicting valence and arousal [75].

Several studies involve a semisupervised approach as well. This approach may be helpful if both labelled and unlabelled samples are available. In [64], for example, a model is first trained on the basis of labelled feature vectors and is then used to classify the unlabelled examples. The samples classified with high confidence are assigned the labels, added to the labelled data set, and the model is retrained. The method can only be applied if the labelled and unlabelled examples are from the same distributions, i.e., from the same user. The problem of unlabelled samples appears if users do not fill in a questionnaire or if the system collects data continuously but it seldom asks for a self-report to avoid disturbing the user.

The unsupervised approach is also sometimes applied as a stage preceding the supervised training phase. The unsupervised methods are applied for representation learning, e.g., in [49], where a variational autoencoder was used to infer low-dimensional embeddings of the input heat-maps presenting touchscreen typing characteristics, and then fully connected layers were implemented as a classifier. Another example is an LSTM-based encoder-decoder used to extract representative features for a typing session [109]. It transforms sequences of typing data into vectors of eight values which are then input to a neural network which was trained to classify them into several emotional states.

One of the factors influencing the choice of the applied method is the form of the target variable. First of all, the applied classification models may be binary [37,40,49,53,65,74,76,116] when the aim is to detect an emotional state, e.g., stress [37,76,116], boredom [53], anger [40,74], happiness [40,74], or sadness [74]. Even if the users report different emotional states, eventually, the binary models are often trained separately for each emotion, either training to discriminate between a selected state and all others or by selecting two states and discriminating between them, e.g., anger vs. neutral or happiness vs. neutral [40]. Sometimes, binary models are trained for each pair of emotions present in the data set [48], which entails deciding how to make the final decision on the basis of the outputs of a set of binary classifiers. It is also possible to cluster emotions into groups and then train a binary classifier, e.g., pleasant vs. unpleasant or activated vs. deactivated [45,54]. The other possibility is to train a multiclass classifier [40,45,48,51,55,74,82,86,108], which requires collecting a sufficient number of samples for each emotional state, which is often problematic. The task also becomes multiclass if a model is supposed to recognise several levels of an emotional state, e.g., stress [63,69,99], happiness [115], depression and anxiety [99], displeasure, tiredness, and tensity [58]. The above also applies to other emotional models, e.g., PAD. It is possible to recognise several levels, usually three or five, for each dimension under consideration, as, for example, in [49,73,82] or to distinguish only two states, making the problem binary [43,67,75,86].

In the case of recognising several emotional states by applying a multiclass approach, a designer often has to face the problem of class imbalance that usually happens in real-world data. Some emotions are experienced more often than others, and therefore the number of gathered samples of selected classes may be much lower. Some methods are sensitive to such an imbalance, and it may have a negative influence on the final model’s efficiency. To address this problem, the Synthetic Minority Over-sampling Technique (SMOTE) is often applied [119]. The algorithm oversamples the minority class by creating “synthetic” examples. The majority class, at the same time, may be undersampled by randomly removing samples. It has been applied, for example, in [109], where among four classes, there were only 7% of *sad* examples, whereas 51% of *relaxed* ones. In [69], the reported five stress levels were highly imbalanced. The higher the level of stress, the lower the number of examples that were observed. In this case, the levels were first merged to three and then SMOTE was applied. Applying this technique may lead to higher classification accuracy as compared to a model trained on the basis of imbalanced data [55,87].

One of the main decisions which has to be made while designing an emotion recognition system is whether to train a general [37,40,67,68,74,82,99] or personalised [50,55,58,78,79,82] model. A general model is trained on the basis of training samples gathered from all users and applied for any user, whereas a personalised one is an individual model adjusted to a selected user on the basis of her data and then applied only for that user. It is of great importance especially in the case of behavioural data, often collected via different personal devices. It is obvious that the way people react to some emotional states may strongly vary among users. Therefore, personalised models seem to be the right choice. Numerous studies where both approaches have been tested show that personalised models lead to higher emotion recognition accuracy [41,61,69,75]. However, they are not free from limitations. To train a personalised model for a user, it is necessary to collect enough labelled data from her. Data collection, especially collected during the user’s everyday interaction, is a time-consuming process. Therefore, hybrid approaches are worth investigating.

A simple idea to overcome this problem was implemented in [76], where models were trained to detect stress on the basis of accelerometer data and some additional information. Although general models did not perform well, the creation of separate models for each of several personality types and weather conditions led to higher accuracy. The type of user personality was estimated on the basis of a questionnaire filled in by the users, while the weather parameters were extracted from public sources.

Another hybrid approach was applied in [61], where a regression model was trained on a small set of data from the selected user and a large set from the general population, but an objective function was modified to prioritise reducing residual errors related to the personalised samples above errors related to samples from others. In this way, the trained model was able to take into account both individual characteristics and those typical for all users.

Another idea of incorporating individual and general patterns was proposed in [109], where a deep neural network was applied to solve a multitask problem, in which each task corresponds to recognising the emotions of an individual user on the basis of their typing behaviour. The network consists of two initial layers which are shared layers common for all users and a final layer, responsible for recognising personalised user-specific patters. Data from each user are used to update the weights of the shared layers and the final layer corresponding to that user. The results obtained by the network are better than those obtained by single personalised networks.

Another way to cope with the problem of scarce user data is to apply transfer learning [64]. In contrast to semisupervised, transfer learning can be applied even if the data sets are from different distributions, so it is possible to use models trained from other users. When a new user appears, a tree is built on the basis of his scarce data. Then several models of other users are selected on the basis of a similarity measure which takes into account both the structure of the trees and the predictions. It is possible either to transfer data used for training the selected models and train one tree or to transfer models and create an ensemble. The results show the ensemble approach provides better results.

A similar idea was presented in [63]. Instead of general or personalised models, the authors introduced a similar-users model. In this case, the model was built on the basis of data from users showing similar behaviour. For each user, a behaviour vector was created. These vectors contain, for each feature, the differences of the medians between every pair of recognised stress levels. After clustering the behaviour vectors, the cluster closest to the vector of a given user is selected as a set of users of similar behaviour.

An interesting solution based on transfer learning was also described in [72]. At the start, a general model based only on a small number of labelled samples from several users is available. This model is used to label instances by applying transfer learning, which takes into account the relationship between the input vector and the vectors used to build the general model. When enough data from the user is collected, a personalised SVM model is also trained. Whenever a new sample appears, it is labelled by the personalised model only if it comes from the same feature space, which is estimated on the basis of an anomaly detector. Otherwise, the general model is applied. The results of the labelling are validated on the basis of the contents of messages or social media entries or by asking the user. If the obtained ground truth is different than the inference result, the sample is stored and later used to retrain the general and the personalised model. The main advantage of this solution is that it allows the system to start creating the first version of the general model with only a small amount of data. Due to validation of new samples and retraining, the accuracy rises.

The reviewed methods and extracted features are presented in Table 2 and Table 3 respectively. The second column of Table 2 includes the emotional states recognised together with information on the number of classes, which is not always the same as the number of points on a scale presented to the users while filling in the self-reports. The given number of participants is the number of users whose data was included in the training sets. The number of participants at the beginning of the experiments was often greater. The last column of input information takes into account any data other than the seven previously presented channels. It includes other sensors, e.g., microphone, camera, and other types of information, e.g., phone usage, weather, time, WiFi traffic, etc. The methods and results mentioned in the last two columns of the table apply to the best configuration obtained, although usually the experiments involved more algorithms. The table includes only the studies, where particular recognition results were given; it does not take into account studies on correlations between parameters and emotions if neither a classification nor regression model was trained. The result value is usually the recognition accuracy, unless otherwise stated, e.g., area under ROC curve (AUC), F-measure. The results given are usually average values obtained on the basis of the results for different emotions (if several binary classifiers were tested) or averaged over users in the case of personalised models, etc.

## 5. Conclusions

The large number of the presented research studies confirms the high interest in mobile applications able to sense emotions. The survey also indicates different ideas that may lead to a successful mobile sensing solution. However, there are also some drawbacks and difficulties which cannot be neglected.

### 5.1. Design Guidelines

The reported recognition results confirm that it is possible to recognise emotional states or mood on the basis of data acquired from smartphone sensors. Numerous factors affect the accuracy of such a system. However, it is impossible to indicate the best configuration of the selected sensors, feature extraction procedure, classification method, etc. The presented results are incomparable. Most studies are performed on the basis of individual experiments considering training data gathered specifically for this research. Thus, the results presented in the papers are obtained for different data sets. However, some general tips may be given.

1. Selection of input channels. One of the first decisions a designer has to make up is the selection of input channels. This decision mainly depends on the type of application. If data is supposed to be recorded while user interacts with a specific application designed for this purpose, then touchscreen and inertial sensors should be considered. Otherwise, i.e., if it is supposed to be a continuous sensing application recording data in the background, then inertial sensors, GPS, and Bluetooth might be taken into account.
2. Sensing schedule design. In the case of continuous sensing, devising a compromise between accuracy and energy cost is a challenge. A higher sampling rate obviously leads to more accurate data series, but it drains the battery. It is especially troublesome in the case of GPS sensing. Therefore, an optimal sampling schedule should be applied. It may be based on time of day or events detected, e.g., GPS recording may start when movement is detected [111] or the resource allocation may be adaptive depending on a user’s current priorities [113].
3. ESM schedule design. Much attention should also be paid to interface design to make the data collection process as unobtrusive as possible. This mainly applies to data labelling, which should ensure a compromise between the amounts of obtained labelled samples and possible user fatigue. Several ESM scheduling techniques are presented in Section 4.2.2. A user should always have an option not to send a self-report or even the possibility to initialise the report himself.
4. Data preprocessing. One of the first steps of raw data preprocessing is the removal of incomplete or inaccurate samples, e.g., a session shorter than a predefined minimum time, a sample containing too little data, e.g., a text message shorter than a specified minimum length. Unlabelled samples should be deleted as well if data are collected for training and supervised techniques are to be applied. Another preprocessing step is normalisation, e.g., in the case of spacial touchscreen data, normalisation should be performed if the system if the application is supposed to be independent of different screen sizes. For time-series data, e.g., obtained from accelerometer or gyroscope, noise reduction is usually performed, e.g., by applying a moving average filter to each of the three axes. GPS locations are often clustered to identify a set of regions.
5. Data segmentation. Raw data need to be split into frames. In the case of time series data, they are split using a sliding window of a predefined length. They may be also split into frames of different length by identifying characteristic points in the series, e.g., it is possible to split a sequence of accelerometer values into subsequences of single steps. In some cases data frames are determined by activities performed within a specified application, e.g., data recorded during a typing session may be treated as a sample.
6. Feature extraction. Depending on the input channel, various features may be extracted as it is described in detail in Section 4.3. The selection of features to be implemented also depends on the type of emotional states, that are going to be recognised. A number of insights on the discriminative power of features in the case of different emotions are given in Section 4.4. In general, touchscreen and accelerometer usually provide valuable information on valence and arousal, whereas GPS on valence, e.g., pleasure is positively correlated with location variance. Touch pressure and some gesture characteristics are good predictors of arousal and valence and they turn especially useful in the case of stress detection when motor behaviour becomes less accurate. Bluetooth is especially useful when social interactions, which also correlate with valence, are to be analysed. At this stage, one should not bother on the number of implemented hand-crafted features, because the next stage would reduce their number. However, the complexity of data extraction should be taken into account if inference on new samples is to be performed in real-time.
7. Dimensionality reduction. Among features extracted in the previous stage, there might be a number of irrelevant ones. Moreover, high number of features increase the computational complexity of applied algorithms and the complexity of the models. Therefore, a feature selection procedure should be performed. Features may be filtered independently using information gain of Gini coefficient. However, a better subset of features could be identified if feature dependence was taken into account, e.g., by applying a sequential feature selection procedure. In both cases, one may assign a threshold value for the number of selected parameters. In the case of sequential selection, another criterion applied may be the recognition accuracy of a model trained on the basis of analysed feature subsets. In the case of large amounts of training data, a proper choice is to find personalised feature subsets, as different features prove to have discriminative power for different users [56,57,60,61].
8. Model training. First of all, personal models adjusted for individual users are preferred [41,61,69,75]. Although they require large amounts of data from one user to be trained with high accuracy, it is possible to start with a general model and improve it when more personal samples are collected [72] or to use the knowledge contained among similar users [63,64]. Possible feedback from users on system accuracy is advisable even if the personalised model has already been trained and deployed. This is a good way to continuously validate the system and retrain the models to reduce the error rate.

### 5.2. Privacy

An extremely important issue related to the development of emotion recognition algorithms, which cannot be ignored and must be taken into account, is the privacy concern of smartphone users. There are two opposing goals here. On the one hand, all kinds of algorithms, especially deep neural networks (DNNs), require large amounts of data and continuous user tracking. On the other hand, device users value their privacy and even anonymity very much. Since the data needed to recognise emotions is usually very personal, there are a lot of problems related to obtaining this data, anonymising it, ensuring its safe transmission, and storing it on servers. The design of a real-life mobile sensing application requires special privacy concern. This is one of the main barriers that limits the social acceptance of mobile sensing systems [71]. The basic approach is not to record sensitive information [69]. In [55,78], where emotions are recognised on the basis of typing characteristics, only nonalphanumeric characters are recorded [55,78]. In [50], instead of analysing location on the basis of GPS readings, cell IDs are taken into account. Giving users control of the sensors being used through configuration options is a good strategy [77]. However, the machine learning models then have to be able to cope with the problem of missing data. All users should be aware of the data collected, and it has to be their individual decision on giving any permission, taking into account the trade-off between the benefits they may get from exploring their data and the possibility of sensitive information being revealed [5].

### 5.3. Applications

Emotion recognition methods can be applied in various areas of smartphone use, including mobile video games and the entertainment industry, intelligent toys, toys and robots, in-car board systems, diagnostic tools for therapists and therapeutic applications, intelligent tutoring systems, affect-sensitive systems for customer services, affect-related research (e.g., in psychology, psychiatry, behavioural, and neuroscience) and many others. Some of the application areas related to remote diagnostics, therapy, and science will benefit significantly in the near future due to the emerging need for remote communication between people, which in many cases takes place using smartphones. The basis of these methods is communication, which may be enriched in various ways. In [51] several emotional states are recognised while tweeting and this information is added to the tweets. Several sensors are involved in this application, i.e., touchscreen, accelerometer, light, and GPS. The average recognition accuracy obtained in this application is 67.52%. Another application is an affective telepresence system proposed in [84]. In this system, a virtual character adjusts its facial expression and body language depending on affective states detected on the basis of data from the accelerometer, GPS, and some additional information on phone usage. The system detects valence, arousal, and their dynamic changes. Emotion recognition was also implemented in a stress-aware virtual environment for conflict resolution, in which information on detected stress may be used by the mediator to make better decisions [37]. The mediator may, for example, advise rethinking a decision when the participant is stressed or take a break. Stress, in this case, is recognised on the basis of acceleration, intensity of touch, accuracy of touches, and amount of movement taken from camera. The obtained detection accuracy is 78%.

Numerous applications relate to mood, which affects our daily behaviours, so accurate estimation of mood can be beneficial for mental and physical health [22,120]. Monitoring mental health may be associated with providing early interventions, e.g., to reduce depressive symptoms and improve stress coping. In [75] for example, where the mood is estimated on the basis of location data, an emotion-aware chatbot suggests some activities depending on the mood. One of the main advantages of such an application is the possibility to reduce the burden on participants present when they are supposed to fill questionnaires.

Particular attention is paid to stress monitoring, because long exposure to stress is associated with various diseases, e.g., coronary artery disease, cardiovascular disease, diabetes, and mental disorder [63,69]. Therefore, numerous studies aim at stress detection. In [76] stress was detected on the basis of Bluetooth data, sms and call logs, and weather information achieving accuracy of 72%. It is also possible to infer stress level on the basis of accelerometer data [63] or accelerometer data together with location changes and phone usage [64]. Both mentioned applications achieved accuracies of around 71% in the case of personalised models. Touchscreen patterns are also stress indicators. In [69] models trained on the basis of different gesture parameters achieved accuracy of around 80% in predicting stress level.

Recognition of emotional states can also be applied in health care to support experts in monitoring patients [50,93]. In [93] mood, anxiety, and depression are recognised on the basis of data read from GPS, accelerometer, and gyroscope. In this case, the automatic mood recognition was performed in order to understand how breast cancer patients responded to a behavioural intervention. Monitoring mood becomes a matter of concern especially among patients suffering from mental disorders. Early intervention in these cases is essential for successful therapy. Therefore, numerous applications are proposed to monitor mood on the basis of smartphone sensors in order to detect symptoms of various mental disorders, e.g., depression [121], bipolar affective disorder [122].

It has been observed that mobile mood-monitoring applications are positively perceived by youth [123]. Numerous users are interested not only in monitoring their behavioural patterns and getting to know how it affects their well-being [77] but also in sharing their mood with others [57,61,114].

An interesting idea of integrating an emotion-recognition module in a cyberbullying detection system was presented in [99]. The idea is based on the assumption that cyberbullying events may convey negative emotional states, e.g., anxiety or stress. In the presented study, the negative states are recognised only on the basis of touchscreen data.

### 5.4. Future Trends

Although it is difficult to accurately predict the directions of further development in the field of mobile affective computing, it is possible to indicate several trends and development tendencies that have started to appear in recent years.

Undoubtedly, one of the clear trends in recent years is the development of personal systems monitoring widely understood user health. This is clearly visible on the example of training watches and smart watches, the functionality of which is beginning to be more and more similar and focused on the user’s health care. In both groups of devices, sensors are already present or are beginning to appear, enabling, among others, the measurement of physiological parameters such as the measurement of heart rate (HR) and its variability (HRV), blood oxygen saturation, body and ambient temperature, humidity, and even electrocardiogram (ECG) and blood pressure (BP). Although these sensors are usually not very accurate, some of them are gaining certification from national health agencies, including the FDA. This basic functionality allows for the implementation of many important goals, such as: analysing the user’s fitness, current fatigue, sleep and rest comfort, and even detecting disturbances and irregularities in the heart rate.

As these personal devices tightly integrate with smartphones into a single ecosystem, this enables them to be used to recognise the user’s emotions. In fact, access to the user’s current physiological parameters takes the recognition algorithms to a completely new level, in which indirect inference based on external observations of the users or their actions can be confronted and supplemented by direct reactions and the state of his body. This allows for the development of a whole group of new, multimodal algorithms for learning and fusion of data or classification results.

Another clear trend is the incorporation of new, advanced sensors and Internet of things (IoT) devices into the smartphone ecosystem, as is the case with thermal sensors. They are absent from the mainstream of the market and are found only in specialised smartphones, e.g., the CAT series from Caterpillar Inc. They are also available as external FLIR Systems smartphone modules, but they are not very popular due to their relatively high cost. Meanwhile, thermal imaging cameras are a great nonobstructive and noninvasive-source of information about some human physiological parameters, allowing not only the measurement of the temperature of the face or body but also easy and reliable measurement of the heart rate, respiratory frequency, etc. Paradoxically, the increased risk of viral infections may increase the popularity and the presence of such sensors in smartphones in the near future.

To sum up, the review presented in the article shows that recognising the emotions of smartphone users with the use of built-in sensors is feasible and has attracted the attention of many researchers in recent years. Although it is a relatively new field of research in the area of affective computing, it has already noted numerous successes, and the reported effectiveness of many methods and algorithms already allows their implementation in utility applications. Undoubtedly, mobile affect-aware applications are becoming common in various areas of our everyday life.

## Figures and Tables

**Table 1:** Low-level features extracted on the basis of accelerometer time series data.

**Feature**	**Equation**	**References**
mean	$\mu =\frac{1}{n}\sum_{i=1}^{n}x_{i}$	[43,48,60,63,68,73,74,82]
median	$median=\frac{1}{2}\left(x_{\left\lfloor (n+1)/2\right\rfloor }+x_{\left\lceil (n+1)/2\right\rceil }\right)$	[48,63,68]
maximum, minimum	$max=\max_{i}\left(x_{i}\right)$, $min=\min_{i}\left(x_{i}\right)$	[43,48,63,68,82]
range	range=max−min	[63]
interquartile range	$IQR=Q_{3}-Q_{1}$, where $Q_{3}$ and $Q_{1}$ are the third and the first quartiles	[48]
variance	$var=\frac{1}{n}\sum_{i=1}^{n}{\left(x_{i}-\mu \right)}^{2}$	[60,63]
standard deviation	$\sigma =\sqrt{\frac{1}{n}\sum_{i=1}^{n}{\left(x_{i}-\mu \right)}^{2}}$	[40,43,48,63,68,73,82]
mean absolute deviation	$MAD=\frac{1}{n}\sum_{i=1}^{n}\left|x_{i}-\mu \right|$	[48]
skewness	$s=\frac{E{\left(x-\mu \right)}^{3}}{\sigma^{3}}$	[40,48,73]
kurtosis	$k=\frac{E{\left(x-\mu \right)}^{4}}{\sigma^{4}}$	[40,48,73]
root mean square	$RMS=\sqrt{\frac{1}{n}\sum_{i=1}^{n}x_{i}^{2}}$	[48,63,110]
energy	$eng=\sum_{i=1}^{n}x_{i}^{2}$	[48]
power	$pow=\frac{1}{n}\sqrt{eng}$	[48]
magnitude	$mag=\frac{1}{n}\sum_{i=1}^{n}\sqrt{x_{i}^{2}+y_{i}^{2}+z_{i}^{2}}$	[63]
signal magnitude area	$SMA=\frac{1}{n}\sum_{i=1}^{n}\left(\right|x_{i}\left|+\right|y_{i}\left|+\right|z_{i}\left|\right)$	[48,63]

**Table 2:** Studies on smartphone emotion recognition. m/f—males/females, T—touchscreen, A—accelerometer, G—gyroscope, M—magnetometer, P—GPS, B—bluetooth, L—light, O—other information.

**Reference, Year**	**Emotions Recognized**	**Elicitation**	**Participants**		**Input Channel**								**Methods**	**Results**
			**Num (m/f)**	**Age**	**T**	**A**	**G**	**M**	**P**	**B**	**L**	**O**		
[49], 2020	valence, arousal, dominance (3 levels); stress, anger, happiness, sadness, surprise (binary)	yes	70 (35/35)	18–31	x								deep learning (variational auto-encoder + fully connected layers); general model	ACC/AUC 67%/0.84 (valence), 63%/0.82 (arousal), 65%/0.82 (dominance); AUC: stress 0.8, anger 0.84, happiness 0.88, sadness 0.87, surprise 0.76
[48], 2020	sadness, happiness, anger, surprise, disgust, fear	no	40 (26/14)	avg 25.2		x	x						SVM	95% (pair-wise), 86.45% (multiclass)
[99], 2020	stress, anxiety, depression (5 levels)	no	115 (100/15)	avg 19.8	x								random forest (general)	stress and anxiety 73.4%, depression 74.1%
[112], 2019; [80], 2020	valence, arousal (5 levels)	no	50			x	x		x		x	x	classifier fusion (neural networks, decision trees)	71.67%, 72.37%
[43], 2019	valence, arousal (binary); affect (positive/negative)	yes	41 (33/17)	avg 24.42	x	x	x					x	SVM, personalised	browsing: 81% valence, 85% arousal, 81% affect; chatting: 69% valence, 72% arousal, 62% affect
[46], 2019	valence, arousal (binary)	yes	33 (17/16)	avg 24.19	x	x							naive Bayes, SVM (general, 4 classes as 4 combinations of valence/arousal)	86.6% (naive Bayes), 83.21% (SVM)
[75], 2019	valence, arousal (continuous)	no	39 (32/7)						x			x	personalised regression (random forest for valence, Ada boost for arousal)	pleasure: 82.2%, arousal: 65.7%
[109], 2019	happy, sad, stressed, relaxed	no	24 (20/4)	avg 23.3	x								multitask neural network (multiclass, first layers shared, then personalised layer)	AUC 84%
[78], 2019; [55], 2019	happy, sad, stressed, relaxed	no	22 (20/2)	24-33	x								random forest (personalised, multiclass)	AUC 78%, 73%
[79], 2018	happy, sad, stressed, relaxed	no	15 (12/3)	24–33	x								deep neural network (personalised, multiclass)	80%
[69], 2018	stress (5 levels)	yes	13 (7/6)	22–32	x								decision tree, k-NN, Bayesian network, SVM, neural network	F-measure, 5-class: individual 79–87% (swipe), 77–81% (scroll); global 75–92% (swipe), 67–78% (scroll)
		no	25		x	x						x		F-measure, 3-class: individual 86–88%, global 63-83%
[54], 2018	excited, cheerful, relaxed, calm, bored, sad, irritated, tense, neutral	no	8 (2/6)			x				x	x	x	random forest	pleasant(excited, cheerful, relaxed, calm)/unpleasant(bored, sad, irritated and tense) 71%, activated(excited, cheerful, irritated, tense)/deactivated(relaxed, calm, bored, sad) 78.3%
[60], 2018	compound emotions (combination of sadness, anger, surprise, fear and disgust)	no	30 (13/17)	18–30		x	x	x	x		x	x	personalised factor graph	76%
[45], 2018	excitement, relaxation, boredom, frustration	yes	20 (10/10)	avg 34	x								SVM, naive Bayes, random forest, logistic regression	4-classes: 67.5% (svm, naive Bayes); 2-classes (excitement + relaxation vs. boredom+frustration): 78.75% (logistic regression), 77.5 (random forest)
[88], 2017	happy, stressed, sad, relaxed	no	22 (20/2)	24–33	x							x	random forest (personalised, multiclass)	AUC 84%
[87], 2017	happy, stressed, sad, relaxed	no	22 (20/2)	24–33	x								random forest (personalised, multiclass)	AUC 73%
[108], 2017	anger, disgust, happy, sad, surprised, fear, neutral	no			x	x			x		x	x	naive Bayes (multiclass)	72%
[67], 2017	valence, arousal (binary)	no	18000			x						x	deep neural network of stacked restricted Boltzmann machines	68% (valence)
[44], 2017	valence, arousal (binary)	yes	29 (29/0)	19–24	x								k-NN, SVM, naive Bayes, decision tree	kNN 94.57%, SVM 96.75%, decision tree 96.4%, naive Bayes 88.4%,
[74], 2017	happy, sad, angry, neutral	no	3		x	x							decision tree (multiclass, general), multi-response linear regression (binary, general)	decision tree: F-measure 0.902, AUC 0.954; regression: F-measure 0.896, AUC 0.851
[73], 2016	valence, arousal (3 levels)	no	10 (3 provided enough data)			x							multilayer perceptron, SVM	arousal 75% SVM, valence 50.9% MLP
[64], 2016	stress (3 levels)	no	30 (18/12)	avg 37.46		x						x	decision trees + transfer learning (personalized)	71.58%
[68], 2016	affect (positive, neutral)	yes	55		x	x	x						SVM (general) for classification and regression	regression (7 point scale): RMSE 1.33; classification (binary): 87.3% (labels on the basis of two elicited states), 89.1% (labels from self reports)
	valence, arousal, affect	no	120		x	x	x						SVM (general) for classification and regression	regression (7 point scale): affect RMSE 1.32, valence RMSE 1.61, arousal RMSE 1.88; classification (binary): affect 69%, valence 81.7%, arousal: 67.5%
[40], 2016	happy, angry, neutral	yes	59 (27/32)			x							SVM (general)	anger(binary): 90.03% (wrist), 90.31% (ankle); happiness(binary): 89.76% (wrist), 87.65% (ankle); happy/angry: 87.1% (ankle); happy/angry/neutral: 85/78/78% (ankle)
[41], 2016	positive, negative, neutral	yes	24 (12/12)	21–25	x								random forest	85.1% (personalised), 78.8% (general)
[63], 2016	stress (3 levels)	no	30 (18/12)	37.46		x							naive Bayes, decision tree (general, personalised, based on similar users data)	general: 52% (naive Bayes), 50% (decision tree); personalised 71%; based on similar users: 60% (naive Bayes), 55% (decision tree)
[72], 2015	sad, happy, angry, content, energetic, tense	no	10 (6/4)	20-40		x			x	x		x	transfer learning (general) + SVM (personalised)	75% (general), accuracy rises after a few days due to validation and re-training
[82], 2015	(1) valence, arousal (5 levels); (2) happiness, sadness, fear, anger, neutral	no	12 (7/5)			x	x	x	x	x	x	x	random forest	general: 65.91% (discrete emotions), 72.73% (pleasure); personalised (one user only): 70.00% (discrete emotions), 79.78% (pleasure)
[53], 2015	boredom (binary)	no	54	21–57							x	x	random forest (general)	82.9%
[50], 2015	mood (5 levels)	no	9 (5/4)	21–27		x					x	x	naive Bayes (personalised)	76%
[39], 2014	stressed, excited, neutral	no	20 (15/5)	21–30		x							decision trees (multiclass)	71% (cross validation), 58% (test set)
[76,116], 2014	stress (binary)	no	117							x		x	random forest, GBM—generalised boosted model (general)	72.51, 72.28% (random forest)%, 71.35% GBM
[115], 2013	happiness (3 levels)	no	117							x		x	random forest (general)	80.81%
[61], 2013	valence, arousal (5 levels)	no	32 (21/11)	18–29					x			x	multi-linear regression (personalised, general, hybrid)	93% personalised, 66% (general), 75% (hybrid, after 30 days)
[51], 2012	happiness, surprise, anger, disgust, sadness, fear, neutral	no	1 (1/0)	30	x	x			x		x	x	Bayesian network (multiclass)	67.52%
[58], 2012	displeasure, tiredness, tensity (5 levels)	no	15			x			x			x	factor graph (personalised)	52.58% (displeasure), 45.36% (tiredness), 47.42% (tensity)
[86], 2012	excited, relaxed, frustrated, bored; arousal, valence (2 levels)	no	15 (9/6)	18-40	x								SVM (general), discriminant analysis (personalised)	general: 88.7% (arousal), 86% (valence), 77% (4 emotions); personalised: 89% (arousal), 83% (valence), 76.4% (4 emotions)
[70], 2012	positive, negative, neutral	no	30						x			x	dynamic continuous factor graph	F-measure 53.31%
[37], 2012	stress (binary)	yes	19	20-57	x	x						x	decision tree (general)	78%


**Table 3:** Features extracted in the reviewed studies.

**Reference, Year**	**Input Channel**	**Features**
[49], 2020	Touchscreen	heat maps of pressure, down-down speed, up-down speed
[48], 2020	Accelerometer Gyroscope	features calculated on the basis of x, y and z sequences: mean, median, standard deviation, max, min, index of max/min, skewness, kurtosis, entropy, root mean square, energy, power, mean absolute deviation, interquartile range, signal magnitude area, zero crossing rate, slope sign change, waveform length; FFT coefficients and their, mean, max, magnitude, energy, band power of signal; sum of squares and sum of absolute values of wavelet transform coefficients; additionally step length and step duration calculated on the basis of accelerometer x series
[99], 2020	Touchscreen	features describing swipes: length, speed, relation between distance and displacement, pressure variance, touch area variance, direction, variance of the angle between points and axes; features calculated for all pairs of consecutive points or all pairs between the starting/ending point of a swipe and any other extracted for eight predefined directions: percentage of touches in each direction, variance of the direction of the vector determined by the mentioned pairs of points
[112], 2019; [80], 2020	Accelerometer	shaking time, severity of shaking, times of shaking, time of portrait orientation, landscape orientation, times of exchanging orientation, step count, difference between average and largest speed
	Gyroscope	rotation time, mean angular velocity
	GPS	entropy
	Light	state (no use/indoor/outdoor)
	Other	network speed, strength of signal
[43], 2019	Touchscreen	touch area, maximum pressure, pressure, hold-time, distance between start and end position, speed, number of touches outside/inside keyboard layout, number of spacebar/send/change language/change number, duration since last press
	Accelerometer	values of x, y, z
	Gyroscope	values of x, y, z
	Other	response time
[46], 2019	Touchscreen	typing speed, backspace frequency, max number of characters without pressing delete for a second, touch count
	Accelerometer	device shake frequency
[75], 2019	GPS	mean an standard deviation of latitude and longitude
	Other	average distance from work, distance from home, time of the day, day of week
[109], 2019	Touchscreen	sequence of vectors containing: intertap duration, alphanumeric (1/0), special characters (1/0), backspace (1/0), touch pressure, touch speed, touch time
[78], 2019; [55], 2019	Touchscreen	see[87]
	Other	last ESM response
[79], 2018	Touchscreen	mean ITD (intertap distance), mean nonoutlier ITD, i-th percentiles of ITD (i = 25, 50, 75, 90), mean and standard deviation of word completion time, session duration, sum and number of ITDs greater than 30s, session duration-pause time, session duration/number of characters, session duration/number of words, percentage of backspace, percentage of nonalphanumeric characters
[69], 2018, solution 1	Touchscreen	tap features: mean pressure, size, movement; scroll/swipe features: mean pressure, size, delta, length; typing features: pressure, tap size, tap movement, tap duration, pressure/size, tap distance, wrong words/all words, back/all digits
[69], 2018, solution 2	Touchscreen	number of touches; minimum, maximum, range, mean, median, variance, standard deviation of touch intervals; session duration
	Accelerometer	scores for various activities (Google API)
	Other	frequency and percentage of time of different application categories, screen features (duration and number of events for differents states: on, off, unlocked)
[60], 2018	Accelerometer	mean and variance of x,y,z; step count
	Gyroscope	mean and variance of x,y,z
	Magnetometer	mean and variance of x,y,z
	GPS	longitude, latitude, altitude
	Light	mean, variance, dark ratio, bright ratio, dark to bright ratio
	Other	application usage (duration for various categories), screen (on ratio, off ratio, Sleeping Duration, Usage Amount), call frequency and duration for each contact person, sms frequency of each contact person, microphone (mean, variance, noise ratio, silence ratio, noise to silence ratio), WiFi (frequency of the top N occurred IDs)
[45], 2018	Touchscreen	touch pressure, touch duration, time between subsequent touches
[88], 2017	Touchscreen	see[87]
	Other	working hour indicator, persistent emotion
[87], 2017	Touchscreen	mean session ITD (intertap distance), refined mean session ITD, percentage of special characters (nonalphanumeric), number of backspace or delete, session duration, session text length
[108], 2017	Touchscreen	typing time, typing speed, key press count, touch count, backspace count
	Accelerometer	device shake count
	GPS	latitude, longitude
	Light	illuminance
	Other	time zone, discomfort index, weather attributes from OpenWeatherMap API
[67], 2017	Accelerometer	DTW distance between the accelerometer readings during the observation interval and the average readings
	Other	DTW distance between microphone readings during the observation interval and the average microphone readings, difference between the number of messages (calls) exchanged during the observation intervals and the average number of messages (calls)
[44], 2017	Touchscreen	number of touch events (down, up, move), average pressure of events
[74], 2017	Touchscreen	average time delay between typed letters, number of backspaces, number of letters
	Accelerometer	average acceleration
[73], 2016	Accelerometer	mean, standard deviation, standard deviation of mean peak, mean jerk, mean step duration, skewness, kurtosis, standard deviation of power spectral density
[64], 2016	Accelerometer	percentage of high activity periods
	Other	location changes (on the basis of WiFi access points, google map locations, cellular towers), conversation time (microphone), parameters form call and sms logs, number of applications used and duration for selected categories of applications
[68], 2016	Touchscreen	finger speed, speed normalised by task difficulty, precision precision normalised by task difficulty, pressure, pressure decline, difference in angle between fingers and centroid at the beginning and end of interaction, angle between horizontal line and line intersecting centroid and tap, approach direction, tap movement, distance between two fingers
	Accelerometer	horizontal and vertical acceleration, difference in aggregated acceleration
	Gyroscope	rotation around x, y, z axis, difference in aggregated rotation
[40], 2016	Accelerometer	standard deviation, kurtosis, skewness, correlation coefficient (for every two axes), FFT coefficients, power spectral density
[41], 2016	Touchscreen	features describing strokes: mean, median, max, min, variance of length, time, pressure and speed of strokes
[63], 2016	Accelerometer	for x, y, z: mean, std, max, min, median, range, absolute value, variance; variance sum, magnitude, signal magnitude area, root mean squared, curve length, non linear energy, entropy, energy, mean energy, standard deviation of energy, DFT (Discrete Fourier Transform), peak magnitude, peak magnitude frequency, peak power, peak power frequency, magnitude entropy, power entropy (for each parameter min, max and mean calculated on the basis of 2 h period)
[72], 2015	Accelerometer	series of state values (run/walk/silence)
	GPS	visited locations, time at locations
	Bluetooth	number of Bluetooth IDs, IDs seen for more than a predefined time, maximum time for an ID seen
	Other	parameters from call and sms logs, number of Wifi signals, content features extracted from text and emoticons
[82], 2015	Accelerometer Gyroscope Magnetometer	for each axis: maximum, minimum, mean, standard deviation, wave number, crest mean, trough mean, the maximum difference between the crest and trough, the minimum difference between the crest and trough; additionally periods of steady/slow/fast on the basis of accelerometer
	GPS	number of locations, entropy (time in different locations)
	Bluetooth	number of connections
	Light	proportion of time for not used, used indoors, used outdoors
	Other	call and message log parameters, number of WiFi connections, application usage time, time of light and dark screen, times of unlocking screen, number of photos, proportion of time in various modes
[53], 2015	Light	ambient light
	Other	connected to headphone or bluetooth, charging, day of week, hour, screen covered or not, ringer mode, average battery drain, battery change during the last session, bytes received/transmitted, time spent in selected applications or sessions, number of notifications, name/category of app that created last notification, number of apps used, number of phone unlocks, time since user last opened notification centre, time since last phone unlock, screen orientation changes, category/name of app in focus prior to probe and name of the previous app, name/category of app used most often
[50], 2015	Accelerometer	activity from Google Activity Recognition Api
	Light	ambient light
	Other	noise, message history, call history, connectivity type (WiFi, mobile, none), calendar (number and type of appointments), daytime, day type (weekday/weekend), location (cell ID)
[39], 2014	Accelerometer	raw values of x, y, z
[115], 2013;[76], 2014; [116], 2014	Bluetooth	general proximity information, diversity (entropy of proximity contacts, the ratio of unique contacts to interactions, the number of unique contacts), regularity (mean and variance of time elapsed between two interaction events)
	Other	general phone usage (number of outgoing, incoming and missed calls, number of sent and received sms), diversity (entropy of contacts, unique contacts to interactions ratio, number of unique contacts), regularity (average and variance of time elapsed between two calls or two sms or call and sms); second order features (selected statistics calculated for each basic feature); weather parameters (mean temperature, pressure, total precipitation, humidity, visibility, wind speed metrics)
[61], 2013	GPS	number of visits in selected locations
	Other	emails (number of emails, number of characters), sms (number of messages, number of characters), calls (number of calls, call duration); number of visits on website domains; application usage (categories, number of launches, duration)
[51], 2012	Touchscreen	typing speed, maximum text length, erased text length, touch count, long touch count, frequency of backspace, enter and special symbol
	Accelerometer	device shake count
	GPS	location (home, work, commute, entertain etc.)
	Light	illuminance
	Other	time, weather, discomfort index calculated as 0.4(Ta+Tw)+15, where Ta is dry-bulb temp., Tw is wet-bulb temp.
[58], 2012	Accelerometer	activity (proportion of sitting, walking, standing, running), micromotion (picking a phone and doing nothing for longer than a few seconds)
	GPS	location
	Other	communication frequency (sms, calls)
[86], 2012	Touchscreen	features describing strokes: mean, median, maximum and minimum values of the length, speed, directionality index (distance between the first and the last point of a stroke), contact area
[70], 2012	GPS	location (region id)
	Other	sms text, calling log
[37], 2012	Touchscreen	mean and maximum intensity of touch, accuracy of touches-relation between the touches on active versus passive areas
	Accelerometer	acceleration
	Other	amount of movement (taken from camera)
